# Vespucci: a system for building annotated databases of nascent transcripts

**DOI:** 10.1093/nar/gkt1237

**Published:** 2013-12-04

**Authors:** Karmel A. Allison, Minna U. Kaikkonen, Terry Gaasterland, Christopher K. Glass

**Affiliations:** ^1^Department of Cellular and Molecular Medicine, University of California San Diego, 9500 Gilman Drive, La Jolla, CA 92093-0651, USA, ^2^Department of Bioinformatics and Systems Biology, University of California San Diego, 9500 Gilman Drive, La Jolla, CA 92093-0651, USA, ^3^San Diego Center for Systems Biology, University of California San Diego, 9500 Gilman Drive, La Jolla, CA 92093-0375, USA, ^4^A.I. Virtanen Institute, Department of Biotechnology and Molecular Medicine, University of Eastern Finland, P.O. Box 1627, 70120 Kuopio, Finland, ^5^Institute for Genomic Medicine and Scripps Institution of Oceanography, University of California San Diego, 9500 Gilman Drive, La Jolla, CA 92093-0651, USA and ^6^Department of Medicine, University of California San Diego, 9500 Gilman Drive, La Jolla, CA 92093-0651, USA

## Abstract

Global run-on sequencing (GRO-seq) is a recent addition to the series of high-throughput sequencing methods that enables new insights into transcriptional dynamics within a cell. However, GRO-sequencing presents new algorithmic challenges, as existing analysis platforms for ChIP-seq and RNA-seq do not address the unique problem of identifying transcriptional units *de novo* from short reads located all across the genome. Here, we present a novel algorithm for *de novo* transcript identification from GRO-sequencing data, along with a system that determines transcript regions, stores them in a relational database and associates them with known reference annotations. We use this method to analyze GRO-sequencing data from primary mouse macrophages and derive novel quantitative insights into the extent and characteristics of non-coding transcription in mammalian cells. In doing so, we demonstrate that Vespucci expands existing annotations for mRNAs and lincRNAs by defining the primary transcript beyond the polyadenylation site. In addition, Vespucci generates assemblies for un-annotated non-coding RNAs such as those transcribed from enhancer-like elements. Vespucci thereby provides a robust system for defining, storing and analyzing diverse classes of primary RNA transcripts that are of increasing biological interest.

## INTRODUCTION

High-throughput sequencing has opened up a new window into transcriptional biology and the complex regulatory networks that define RNA and DNA interactions. Global run-on sequencing (GRO-seq) (1) is a recent addition to the series of sequencing-based methods that holds particular promise for understanding real-time transcriptional behavior. GRO-seq captures a point-in-time snapshot of active transcription genome-wide and returns data on the position, length and orientation of nascent transcripts.

This sequencing technique is now being used to inspect the nature of transcriptional regulation in a number of experimental conditions (1–4). The capture of nascent transcripts in each of these conditions reveals a variety of RNA species beyond the standard set derived from genes encoding proteins and microRNAs, including enhancer RNA (eRNA), long intergenic RNA (lincRNA) (2) and promoter-associated RNA (1,5). GRO-seq thus offers unprecedented insight into the generation of a vast repertoire of non-coding transcripts that are of potential functional significance.

The data collected, however, are both immense and unique; each experiment yields tens of millions of strand-specific short RNA reads across the entire genome. This new sequencing method presents a new algorithmic challenge, as the peak-calling and exonic RNA identification techniques developed for other sequencing methods do not address the particular output of GRO-seq. Unlike ChIP-seq, peaks are not the primary unit of output, and, unlike RNA-seq, nascent transcripts can be anywhere, so relying on previously annotated regions such as NCBI Reference Sequence (RefSeq) (6) or microRNA genes is insufficient.

To take full advantage of this novel data, regions beyond existing annotations must be considered. Units of transcription must be inferred *de novo* from the short read output of GRO-sequencing experiments. Existing analysis of GRO-seq data relies largely on adaptations of RNA-sequencing analysis techniques, with expression levels calculated from tag counts over gene bodies, promoters or other explicitly defined genomic regions (1–4,7). New transcripts can be identified using software such as Cufflinks (8), but these rely on assumptions optimized for spliced RNA. For example, Cufflinks is optimized for paired-end reads, expects uniform density for a given transcript (whereas GRO-seq can reveal pausing and other biologically relevant deviations from uniformity) and aims to accommodate large gaps (introns) in reads that result from splicing rather than from transcriptional breaks. In short, Cufflinks and similar exon-focused algorithms are not suited to distinguish between the sorts of small and closely spaced regulatory elements that GRO-sequencing reveals.

Hah *et al.* have developed a Hidden Markov Model (HMM) for identification of regions of transcription specifically within GRO-seq data (2). The software demarcates transcripts using a two-state model, calling regions either ‘transcribed’ or ‘un-transcribed’, and thus is able to identify transcripts from GRO-sequencing short reads *de novo*. However, the HMM is optimized to accurately retrieve transcript boundaries as defined by RefSeq, resulting in the loss or merging of many of the shorter, non-coding RNA transcripts that GRO-sequencing reveals. Further, because the software relies on flat files for processing and storage, it is difficult to integrate the called transcripts with other types of genomic data, including expression levels from each individual GRO-sequencing experiment and co-occurring peaks from ChIP-sequencing data.

Here, we provide an algorithm for *de novo* identification of unified transcripts from GRO-seq data, along with an implementation that determines transcript regions, stores them in a relational database and associates them with known reference annotations according to 2D genomic overlap. Crucially, this method captures transcript boundaries as defined by RefSeq while maintaining the ability to identify non-coding RNAs at a high resolution and even retaining information about relative transcript abundance. Further, transcript identification feeds into a database that makes downstream integration of other datatypes feasible.

Using this system, we were able to gain new insight into the types of nascent RNAs being generated inside primary murine macrophages. Although the ENCODE Project has begun the process of characterizing mature RNA species (9), there is little known about the extent and distribution of nascent RNAs, which, unlike mRNAs observed in traditional RNA-sequencing, include a number of transient RNA species that nonetheless play roles in the regulation of gene expression (1,10–13). Of particular interest are the vast numbers of non-coding RNAs recently found to be derived from transcription of active enhancers (14,15). The finding that at least some of these eRNAs contribute to enhancer function provides impetus for developing computational tools to define the sites of initiation of these species and their length. Importantly, while the start and termination sites of transcripts related to mRNA-encoding genes and lincRNAs have for the most part been established by conventional RNA sequencing studies, this information is virtually non-existent for eRNAs. Furthermore, the ENCODE consortium estimates that the human genome contains hundreds of thousands of enhancers (16), the majority of which are selected in a cell-specific manner. Therefore, each GRO-seq experiment in a new cell type results in the identification of tens of thousands of previously unannotated eRNAs that are derived from transcription of cell-specific enhancers. To address this challenge, we developed Vespucci as a computational method to systematically and quantitatively define discreet nascent transcripts from short sequencing reads obtained in GRO-seq experiments. By tuning parameters for specific types of transcripts, Vespucci returns accurate calls for primary mRNAs, while also deconvoluting complex patterns of transcription from enhancer-rich regions of the genome. Using Vespucci, we provide evidence that many nascent mRNA transcripts extend well beyond RefSeq annotated termination sites. In addition, Vespucci predicts approximately twice as many non-coding transcripts as were identified by other systems like the Hah *et al.* HMM. These findings demonstrate the value of Vespucci in integrating disparate data types to characterize the variety of RNA species observed.

The Python and PostgreSQL code, as well as a pre-loaded Amazon AMI, have been made available for implementation and expansion by interested researchers.

## MATERIALS AND METHODS

### Technical details

The current implementation allows sample types and database schema to be split easily by cell type, such that the merging of transcripts is confined to a single cell type. Still, GRO-sequencing runs from multiple cell types can be easily merged together if desired.

The current codebase assumes a PostgreSQL 9.2 database installation; Python 2.7+; and Django 1.2+ with psycopg2 for database access. The codebase is hosted on Github at https://github.com/karmel/vespucci, and includes scripts to build both the transcript and the annotation databases. Instructions are included within the repository. In addition, a pre-loaded Amazon EC2 small instance image is available with instructions at https://github.com/karmel/vespucci.

### Cell culture

Primary cells were isolated from 6 to 8-week-old C57Bl/6 mice. All studies were conducted in accordance with the UCSD Institutional Animal Care and Use Committee. Thioglycollate-elicited macrophages were isolated by peritoneal lavage 3–4 days following peritoneal injection of 2.5 ml thioglycollate. Cells were plated in RPMI medium 1640 and 10% fetal bovine serum, washed after adherence and again fed with fresh medium. The following day fresh medium containing 0.5% fetal bovine serum was added to the cells and serum starvation was carried overnight

### GRO-seq library preparation

Briefly, GRO-sequencing takes advantage of a nuclear run-on reaction to incorporate tagged UTP into ongoing transcript synthesis by RNA polymerase. RNAs that incorporate the tagged nucleotides can subsequently be extracted and sequenced, producing a genome-wide library of nascent RNAs. Thus, in contrast to traditional RNA-sequencing in which mature stable RNAs are collected, GRO-sequencing returns short read data for RNAs in the act of being transcribed.

Global run-on (1) and library preparation for sequencing (17) were done as described. The protocol was performed as described in Wang *et al.* (3)

### Previously published GRO-seq data

Four of the replicates were previously published under GSE48759 (4). The GEO Accession codes are GSM1183906–GSM1183908 and GSM1183914. The MCF-7 GRO-seq data are available under GSE27463 (2), Accession codes GSM678535–GSM678540; and GSE45822 (12), Accession codes GSM1115995–GSM1115998.

### Read mapping and ChIP-Seq data analysis

Reads were mapped to the mm9 genome using Bowtie2 (18) with the default alignment options (specifically, the command bowtie2 –no-unal –x).

H3K4me1 and input data were taken from GSE21512 (7), Accession codes GSM537986 and GSM537988. MCF-7 H3K4me2 data and input were taken from GSE24166 (19), Acession codes GSM594606 and GSM594608. Peaks were called using HOMER (7) using the command findPeaks and the options -nfr -style histone.

### Case study counts

The SQL queries used to generate the counts for the analysis of transcription in macrophages are included as a Supplementary File.

## RESULTS

### Defining a transcript

#### Principles

**Figure 1.**
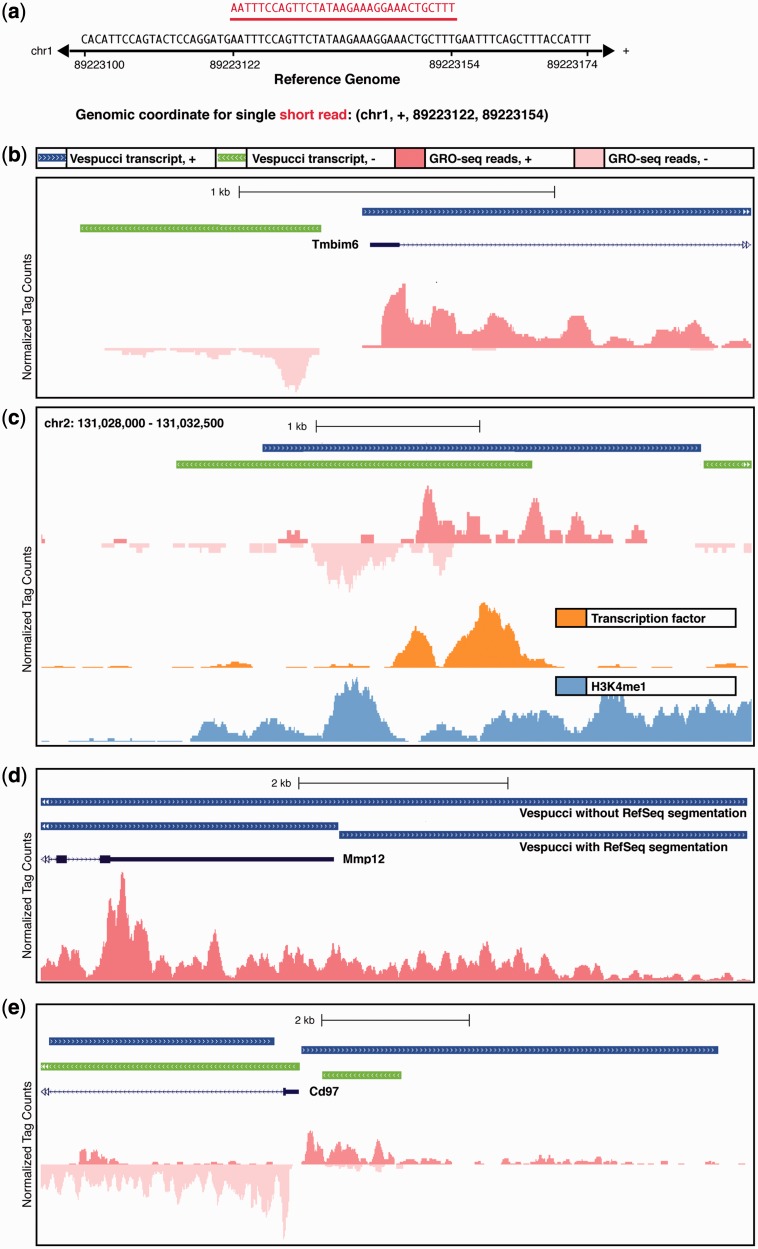
GRO-sequencing reveals transcriptional dynamics in great detail, but can be difficult to interpret. (**a**) Short reads from GRO-sequencing experiments (red) can be mapped back to the reference genome (black) and assigned a genomic coordinate that includes chromosome, strand, starting basepair and ending basepair. (**b**) Promoter-associated RNA (paRNA) overlaps with the 5′ end of the Tmbim6 gene, antisense to the gene itself. The blue bar indicates the transcript that has been identified for Cstb itself, and the leftmost green bar shows the extent of the paRNA. (**c**) Enhancer RNA (eRNA) appears in GRO-sequencing samples (top track) as bi-directional transcripts centered on the binding sites of transcription factors (middle track) and marked by H3K4me1 (bottom track). (**d**) Transcription can continue past the 3′ ends of annotated RefSeq transcripts, making the exact boundary of relevant transcripts difficult to identify. At the Mmp12 locus, manual interpretation could lead to differing interpretations of where to mark transcript boundaries, either including or excluding the run-off at the 3′ end of the gene, and thus it is important to have a consistent algorithmically determined interpretation. Here, we show that Vespucci is able to either respect the RefSeq boundary (lower blue track) or to identify the entire nascent transcript (upper blue track). (**e**) Neighboring transcription regions can have different read densities. Two transcripts are identified along the sense strand, denoted by the blue bars at the top. These two transcripts are close in terms of basepair distance (363 bp apart), but they differ in terms of read-per-basepair densities, and therefore are kept as two separate transcripts.

As with most next-generation sequencing-based methods, GRO-seq relies on short (35–100 bp) reads. For the purposes of this study, we assume each individual read is mapped to the canonical genome of the organism in question with a standard aligner such as Bowtie (20). Any given uniquely mappable read, then, can be placed into 1D space with a definitive coordinate consisting of chromosome, strand, start of read and end of read (Figure 1a).

The location of each short read does not alone describe the relevant units of transcription in the genome; overlapping sets of short reads must be computationally merged so that they represent the extents of biologically relevant transcripts, which here we take to mean linear segments of DNA that are transcribed into continuous RNA sequences by RNA polymerase II (9). Once merged into continuous units, the count of short reads mapping to a given unit (transcript) can be used to approximate the relative expression level of the transcript (21).

A primary challenge in any short read RNA sequencing application is determining how to merge the fragments into unified transcripts. Each type of sequencing presents unique challenges in this regard; in the case of mRNA-seq, for example, methods have been developed that are designed to identify exon junctions (8,22). GRO-seq reads, in contrast, are expected to extend through intragenic regions, and further are expected to exist widely both in intergenic regions and in regions antisense to annotated transcripts (1).

There are numerous patterns of GRO-seq data that are important to identify computationally. For example, promoter-associated RNA transcripts are generated at the promoters of genes, antisense to the genic transcript (1) (Figure 1b). Any algorithm addressing GRO-seq needs to identify these RNAs as distinct units, overlapping with but not part of either genic transcripts or nearby eRNAs. Similarly, eRNAs are generated bi-directionally at enhancers (23), and any algorithm must identify each strand of eRNA as a separate but contiguous unit (Figure 1c).

Notably, some transcripts appearing in GRO-seq data seem ‘obvious’ to separate when viewed in the UCSC browser, as with Figure 1b and c. However, these cases are the minority, and, further, any such ‘obvious’ separation is *ad hoc* and risks inconsistency when performed manually; in Figure 1d, for example, most observers would not separate the transcript, but existing annotation data from RefSeq indicates there is an important boundary corresponding to a coding sequence. Thus, it is useful to have an algorithmic interpretation that provides a standardized analysis and additionally can appeal to existing annotation data if available (Figure 1d).

If there are no existing annotations from which to scaffold the current transcript identification, the algorithm must have a standard means of interpreting the short read data that is likely to reflect the biological reality of the transcriptional data. To this end, the present implementation makes several assumptions based on the expected behavior of RNA polymerase: first, that regions that are tiled without gaps by short reads from a single sequencing run are most likely continuously transcribed; and second, that gaps corresponding with a great disparity of read counts per basepair likely represent breaks in the path of RNA polymerase, with differently regulated transcripts on either side. (Supplementary Figure S1a shows a schematic of how differential density might yield separate transcripts, and Figure 1e shows an example of this in real GRO-sequencing data, where transcription along each strand in the displayed region is split into two separate transcripts due to differential coverage.)

These two principles—that overlapping reads should be merged and that disparity in the density of reads may warrant separation of otherwise close transcripts—motivate the design of the algorithm described later in text.

#### Implementation

Given the size of GRO-seq data sets, with every sample yielding at least tens of millions of reads, any algorithm must be implemented within a framework that is easily maintained and extended, and it must process data quickly enough to be useful in a laboratory setting. Further, the transcript identification system must be architected such that new samples can be incrementally added to the full set of data without requiring re-processing of all data. To this end, we have developed a Python codebase that processes mapped short read files from GRO-seq experiments into continuous transcript units, determines relative expression levels on a sample-by-sample basis and stores the data in a PostgreSQL relational database that allows for complex coordinate-based queries over the transcriptional data.

The procedure relies on two key parameters:

- DENSITY_MULTIPLIER: scaling factor to relate density to basepairs (see step 4 later in text; default: 10 000). Intuitively, this is the number of basepairs over which density is considered, so that a difference in one tag per DENSITY_MULTIPLIER basepairs equates to a one basepair gap in genomic distance.
- MAX_EDGE: maximum allowed distance in 2D space between proto-transcripts to be stitched together (see step 5 later in text; default: 500).

The selection of values for these two parameters depends heavily on the desired use case. The larger the value of DENSITY_MULTIPLIER, the more density matters as compared with distance in basepairs, and the larger the value of MAX_EDGE, the more likely distant transcripts are to be merged into single units. Thus, if the user desires to focus on large transcripts and genes, he/she might choose a low value for DENSITY_MULTIPLIER and a large value for MAX_EDGE. On the other hand, if the user desires to focus on small transcripts and ncRNA, he/she might choose a high value for DENSITY_MULTIPLIER and a small value for MAX_EDGE. The selection of the default values of these parameters, and the values used for the data in this study, are discussed later in text under ‘Parameter Selection.’

**Figure 2.**
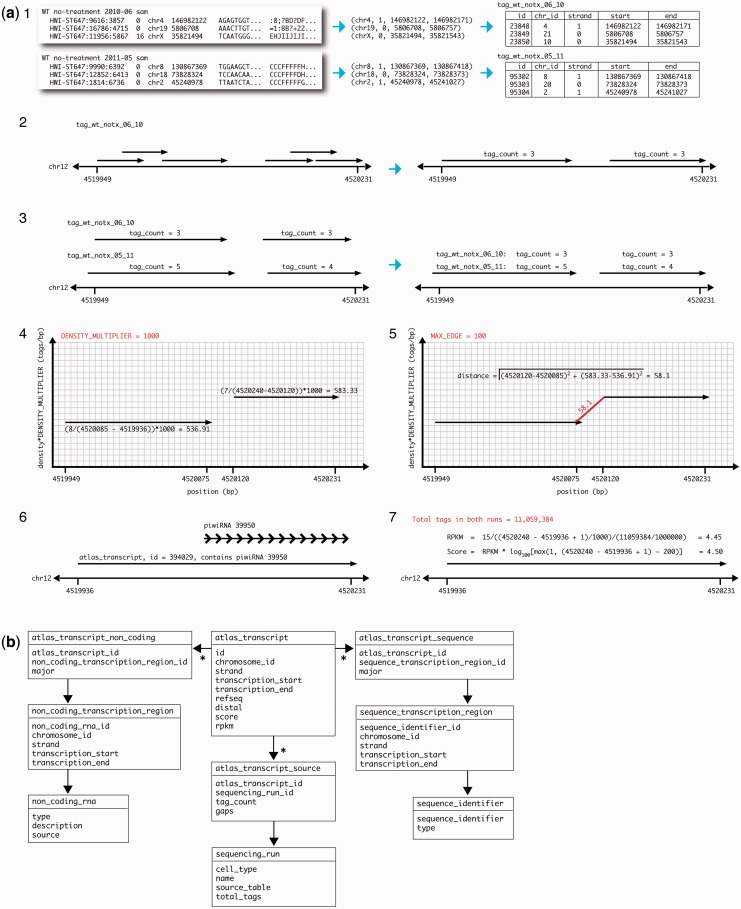
Stepwise procedure for assembly of transcripts by Vespucci. (**a**) (1) Each sample, mapped to the reference genome, is reduced to its genomic coordinates and loaded into a separate database table. (2) Short reads from a single run are merged (separated by chromosome). (3) The merged proto-transcripts from each individual run are merged with proto-transcripts from other runs. The number of tags from each different run is stored. (4) The proto-transcripts from (3) are plotted in 2D space, with location in basepairs along the *x*-axis and the density in tags per basepair along the *y*-axis. The density is scaled according to a parameter, DENSITY_MULTIPLIER, that defines the relationship between the two units of measurement (basepairs and tags per basepair). (5) The proto-transcripts in 2D space are then merged according to a MAX_EDGE parameter that operates as the maximal allowed Euclidian distance from the rightmost edge of each transcript. The merged transcript here is considered a continuous unit of transcription by Vespucci. (6) These transcripts can then be associated with known RNA species from RefSeq and ncRNA databases based on genomic coordinates. (7) Transcripts are then scored according to a custom algorithm or RPKM. (**b**) Database schema showing the Vespucci transcript table structure, major columns and related entities. An asterisk indicates a ‘has many' relationship, and ID fields contain references to related tables.

Once these parameters are set, the processing of reads into transcripts proceeds as follows for each strand of each chromosome (Figure 2a):

1. Given a mapped tag file [in BAM or SAM (24) format], each tag is reduced to its genomic coordinates and loaded into a database table. The tables are designed such that the dataset for each sample is stored in a separate table.
2. Once loaded, the tags from a single sample are merged, but no analysis of density is attempted. Although individual tag boundaries are not maintained in the merged format, the count of reads and number of gaps between the reads that are merged are tracked for expression level comparisons later.
3. The set of unified transcripts from a single sample is then merged with transcripts from all existing samples.
4. Using the stored tag counts and the genomic coordinates of the merged read, each proto-transcript is mapped as a horizontal line segment in 2D space, with the start and end serving as the coordinates along the *x*-axis, and the density (tags in all runs per basepair) as the coordinate along the *y*-axis. Density is scaled by a parameter (DENSITY_MULTIPLIER); a higher multiplier increases the relative importance of density as compared with position.
5. A second stage of merging begins over the 2D space according to the algorithm described later in text and the MAX_EDGE parameter. At this stage, several optimization checks filter out proto-transcripts that are likely noise, such as those that have fewer than one tag per sample on average.
6. Transcripts are associated with annotation databases as described in part 2, later in text.
7. Transcripts are scored. Any scoring algorithm could be implemented here, but currently two are included:
  - A standard reads per kilobase per million tags (RPKM) score assignment
  - A custom, length-sensitive algorithm:

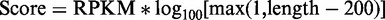
    This custom score has several modifications as compared with RPKM that make it more sensitive to certain kinds of transcripts:
  1. Short transcripts (<200 bp) are set to a score of 0. This reduces noise from overlaps of several reads that get stitched together, and from technical artifacts.
  2. Long transcripts are handicapped. There are many long transcripts with low levels of transcription that are nonetheless interesting (Supplementary Figure S1b). RPKM alone has a tendency to decrease with transcript length (Supplementary Figure S2a), and thus it is difficult to filter out short noisy transcripts without losing long transcripts for which we accept lower levels of transcription. To address this problem, the custom score scales the RPKM by the log of the length of the transcript, thereby leveling out the scores of long transcripts (Supplementary Figure S2b). We use a logarithm with base 100 here to ensure that the score scales only minimally over the extremely wide range of transcript lengths.

The choice of using the RPKM or the custom score depends largely on use case; if transcripts <200-bp long are of particular interest, as might be the case if one were studying pause-release mechanisms using GRO-seq (25), then it would be advisable to use unmodified RPKM instead of the custom score.

When the processing is complete, the derived transcripts can be easily queried, annotated and associated using a relational database for storage (Figure 2b).

#### Algorithm

To stitch together continuous proto-transcripts in a density-aware manner, we first map the proto-transcripts in 2D space: along the *x*-axis is the start and stop, in zero-indexed basepairs of the read, and the *y*-axis represents the mean density of short reads over all samples [Figure 2a(4)]. To relate basepairs distance to read density, the density is scaled by the DENSITY_MULTIPLIER. This graphical representation allows us to define the distance between any two transcripts as simply the Euclidian distance in this density-basepair plane [Figure 2a(5)].

Arguably, it is not necessary to relate density and basepair distance in this manner, and an alternate distance formula could consider separate thresholds for the differences in densities and positions of two proto-transcripts. However, such a distance function would not account for the biologist’s intuition that the closer two transcripts are, the more likely they are to be a single unit, even if there is a density difference that might at a greater positional difference warrant separation of two transcripts. In other words, the difference allowed between densities of two proto-transcripts when merging is dependent on the basepair distance between the two, and thus considering the Euclidian distance is preferable to a binary threshold that treats density and basepairs independently.

With a distance function thus specified, we can define our algorithm for merging proto-transcripts into transcripts:

1. We define a graph in which every node is a proto-transcript, and a pair of nodes is connected by an edge if and only if the distance between the two proto-transcripts is less than or equal to the parameter MAX_EDGE.
2. Connected components in this graph represent merged proto-transcripts.
3. Merged proto-transcripts can then be recast as intervals spanning the minimal basepair start and the maximal basepair end. Overlapping intervals are merged.

At the end of this procedure, we have produced a set of continuous non-overlapping transcripts that can be stored, annotated and so on, as seen in Figure 2a(5)–(7).

A naive algorithm would be quadratic, comparing every node with every other. However, in practice, nodes are ordered, and it is only necessary to consider nodes within a distance of MAX_EDGE. Thus, the algorithm can be practically implemented in linear time with respect to the number of proto-transcripts. In the current implementation, we take advantage of the geometric query space in PostgreSQL to limit the search for neighboring proto-transcripts to a distance of MAX_EDGE.

### Annotating a transcript

#### Using known RefSeq

The procedure described earlier in text can proceed naively—i.e. based entirely on 2D distance between transcripts and without awareness of existing annotations. In practice, it is useful for the implementation of the algorithm to respect the existing boundaries of genes as annotated by RefSeq, as this allows tag counts and computed expression values to be relevant in the context of the existing literature on gene body-based expression comparisons.

Thus, the current implementation of the algorithm makes two important allowances for RefSeq genes. In the first, the allowed distance between proto-transcripts that is traversed during the 2D merging in step 5 can be increased within the boundaries of known RefSeq genes such that gaps are more likely to be covered within genes. This extra allowance increases the likelihood that long low-expression transcripts are recognized as single units rather than a series of small gapped transcripts.

The second heuristic applied to the identification of previously annotated transcripts addresses the continuation of transcription past the traditional transcription termination site. In GRO-seq data, we see clearly that transcription does not always stop at the point corresponding to the annotated gene end, but rather continues on for some distance (Figure 1d). In these cases, we may want to be able to compare GRO-seq expression counts in genes to the measurements made in previous RNA expression studies, and thus force a separation between tags falling within RefSeq boundaries and those that extend beyond the boundaries, even if the signal is continuous according to the general rules of merging outlined earlier in text. Vespucci can be configured either to force the transcript to be segmented according to RefSeq boundaries (lower blue track in Figure 1d), so that comparisons can be made to more traditional expression data, or to assemble the transcript regardless of the annotated RefSeq boundaries (upper blue track in Figure 1d), so that the full nascent transcript can be analyzed. In the case where segmentation along RefSeq boundaries is forced, the post-gene transcript is linked via an index to its preceding gene transcript, and we have used this option in Vespucci in the case study analyses later in text.

#### Annotation from known databases

In addition to segmentation according to known annotations like RefSeq, it is useful to be able to associate the known annotations with the transcripts that overlap in genomic space. Thus, the current implementation includes logic not only to define transcripts according to RefSeq boundaries but also to associate RefSeq identifiers with overlapping transcripts strand specifically. Similarly, we provide logic and data to make associations with non-coding RNA as identified by the Functional RNA Project (26).

#### Arbitrary data types

The representation of transcripts in terms of genomic coordinates gives power beyond associating with existing annotations. Arbitrary data types, such as peaks identified in individual ChIP-sequencing experiments, repeat regions, conservation scores and ESTs, could all be represented in terms of genomic coordinates and used to annotate transcripts, either within the existing framework or *ad hoc*. There are many examples included in Supplemental SQL queries that collectively demonstrate that the power of the current system is its ability integrate expression data across many samples with multiple types of annotative or associative data based on genomic location and distance quickly and easily.

### A case study—transcription in macrophages

Transcriptional profiling accomplished by the ENCODE project has revealed that about three-quarters of the genome is transcribed across fifteen human cell lines (9). Using GRO-seq data from five biological replicates, we analyzed the characteristics of transcription in murine thioglycollate-elicited macrophages using Vespucci, with the intent of characterizing the extent of transcription in a particular primary cell type under unstimulated conditions.

#### Parameter selection

To optimize the selection of the MAX_EDGE and DENSITY_MULTIPLIER parameters, we took advantage of previously published 5′-GRO-seq data (10), which identify nascent RNA with a 5′ 7-methylguanylated cap. The 5′-GRO-seq method thus produces peaks that identify transcription initiation sites of nascent RNAs genome-wide. The data available were in RAW 264.7 cells, which are a macrophage cell line, and thus were expected to be compatible with the primary macrophage GRO-seq data. Because 5′-GRO-seq identifies transcript initiation sites, we would expect transcripts identified by Vespucci to have maximally one 5′-GRO-seq peak; having more than onewould be an indicator that the Vespucci transcript had merged together multiple separate units. Conversely, having zero 5′-GRO-seq peaks within a Vespucci transcript could indicate that noise was falsely assembled into a transcript, that a continuous transcript was divided into many transcripts, or that the two sequencing techniques differ in sensitivity. We desired, therefore, to select parameters that would maximize the rate at which identified transcripts corresponded with exactly one 5′-GRO-seq peak. Further, to avoid advantaging parameters that achieved this higher rate by greatly reducing the total number of transcripts, we added a penalty for the rate at which transcripts were identified with more than one 5′-GRO-seq peak. The resultant metric, which we labeled the Initiation Recapture Rate (IRR), is defined as:

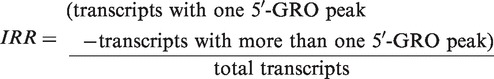



We then tested values of MAX_EDGE in the range of 100–5000 and found that the maximum IRR was achieved at a MAX_EDGE of 500 (Supplementary Figure S1c). Then, holding MAX_EDGE constant at 500, we tested values of DENSITY_MULTIPLIER in the range of 1000–100 000 and found that the maximum IRR was achieved at 10 000 (Supplementary Figure S1d). Thus, we selected a MAX_EDGE of 500 and a DENSITY_MULTIPLIER of 10 000 for the current study and as the default parameters. Notably, as discussed later in text, these values perform well when used with human MCF-7 data as well, implying that the currently selected values are applicable to a variety of experimental data sets.

#### Identification of RNA species

We proceeded to analyze the murine macrophage GRO-seq data with a MAX_EDGE of 500 and a DENSITY_MULTIPLIER of 10 000. Using these values, we see 11% of the sense strand (294 363 940/2 620 345 972 bp) and 11% of the antisense strand (282 540 749/2 620 345 972 bp) being actively transcribed in basal conditions.

These regions of transcription across the genome can then be inspected further. Using the unstimulated data, the total number of transcripts passing the minimal threshold to progress from proto-transcripts into the secondary transcript database is 84 076; of these, 34 743 (41%) had a score (as defined by the custom method described in step 7 of the procedure earlier in text) of at least 1. The score threshold best suited for analysis depends heavily on intent; if the user is interested in transcripts that are transient or have low expression, setting a lower threshold at the risk of introducing some noise may be advised. On the other hand, the HMM described by Hah *et al.* (2) resulted in only 22 893 transcripts in a human cell line; if the user desires a comparable high-threshold analysis with annotated regions making up ∼50% of the transcripts identified, a higher score threshold can be used.

**Figure 3.**
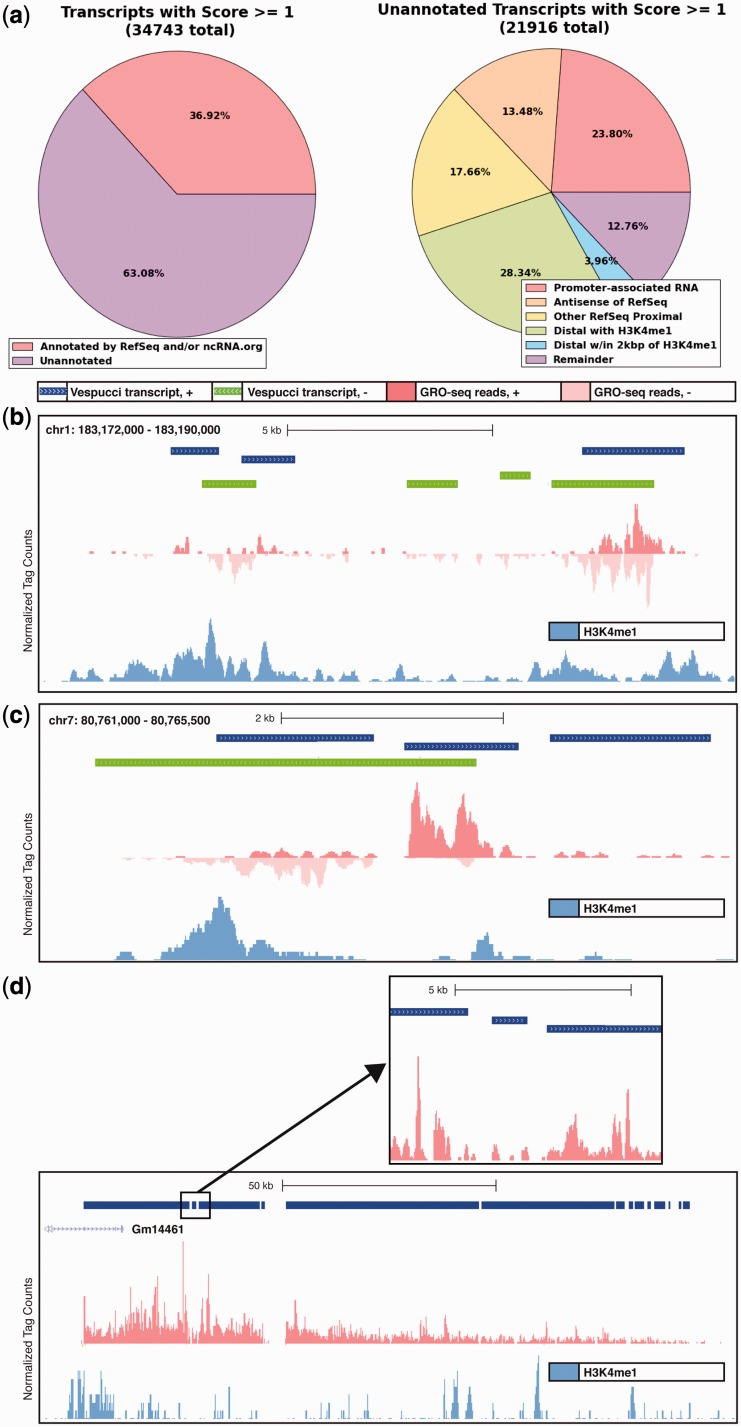
Vespucci enables the identification and quantification of numerous RNA species in macrophages. (**a**) Using a score threshold of 1, the great majority (63%, left panel) of transcripts identified are not associated with known RefSeq genes or ncRNA. Of the unannotated set (right panel), more than one half are proximal to RefSeq genes, with the remainder being distal. (**b**) Transcripts are interspersed not only overlapping with the enhancer histone mark H3K4me1 but also between enhancers, indicating that complex regulatory regions undergo a great deal of active transcription spread over many kilobases. (**c**) Similarly, transcripts can extend a long distance beyond identifying histone marks at enhancers, with this itergenic region showing low levels of H3K4me1 and GRO-seq signal extending along a single strand for >5 kb beyond an identified H3K4me1 peak. (**d**) Vespucci identifies a long unannotated transcript downstream of Gm14461. Vespucci does not merge the entire region, but, with a gap parameter of 100 bp, separates it into several long regions with many shorter regions interspersed throughout. Closer inspection (inset) shows that the boundaries determined by Vespucci reflect real discontinuities in the GRO-seq signal that will require further study to interpret. H3K4me1 is shown on the lower track to indicate that this transcript is methylated at the 5′ end, much as a protein coding gene would be.

Of these ∼35 000 transcripts, only 8742 (25%) overlapped with RefSeq genes such that the gene was at least half transcribed. A further 1573 (5%) overlapped with RefSeq genes (same-strand) but covered less than half of the gene. In all, 8079 (23%) transcripts overlapped with annotated ncRNA (Figure 3a).

The remaining 21 916 transcripts (63%) were not annotated by RefSeq or the ncRNA.org database. These unannotated transcripts comprised a large proportion of the total. Of the unannotated set of transcripts, 12 042 (55%) were within 1 kb of a RefSeq transcript, of which 5216 (43%) were specifically within 1 kb of an active RefSeq transcription start site (TSS), antisense the RefSeq transcript, and thus warranted labeling as promoter-associated RNA. The 2955 transcripts (25%) were antisense of transcribed RefSeq transcript bodies; these include intragenic enhancers and long ncRNA (Supplementary Figure S2c).

Of the 21 916 unannotated transcripts, 9874 (45%) were >1 kb away from any RefSeq transcript, and were thus labeled as *distal transcripts*. It has been established that enhancer elements in the genome are marked by unique histone methylation patterns (27,28)—namely, high levels of H3K4me1 and H3K4me2 but low levels of H3K4me3— and further are actively transcribed, generating transcripts (eRNAs) (23). To assign putative labels for distal transcripts, peaks called by HOMER (7) from H3K4me1 ChIP-sequencing in unstimulated macrophages were loaded into the database and queried. In all, 6211 (63%) of the distal transcripts overlapped with H3K4me1 peaks and were labeled *eRNA*.

The remaining 3663 transcripts—37% of distal transcripts and 13% of all transcripts—had no label. Closer inspection of this subset revealed that 867 of the unannotated distal transcripts were within 2 kb of a H3K4me1 peak. Interestingly, many of these appeared to be regions of transcription between clusters of enhancers (Figure 3b) or enhancer-associated RNA extending far past the range of the histone mark (Figure 3c). Taken together, these results imply that the amount of transcription attributable to enhancers is greater than currently accounted for by analyses looking only at regions directly overlapping associated histone marks.

In addition to these general categories of unannotated transcripts, there were some transcripts in this remainder set that were intriguing anomalies. For example, there was a 100 kb + region directly downstream of RefSeq gene Gm14461 on chromosome 2 that exhibited active transcription, but was entirely unannotated by RefSeq, ncRNA.org or known mouse expressed sequence tags (ESTs) (Figure 3d). Transcription throughout this region was not continuous (Figure 3d, inset), and further there were several stretches of repeats that prohibited unique mapping of tags. Thus, the region was segmented into numerous blocks of transcription. Nonetheless, the identification of such regions demonstrates the importance of closer inspection of GRO-sequencing data for the purposes of finding uncharacterized transcripts, as well as the value of the database described here in building these types of transcripts from merged units.

### Transcription does not stop at RefSeq termination sites

**Figure 4.**
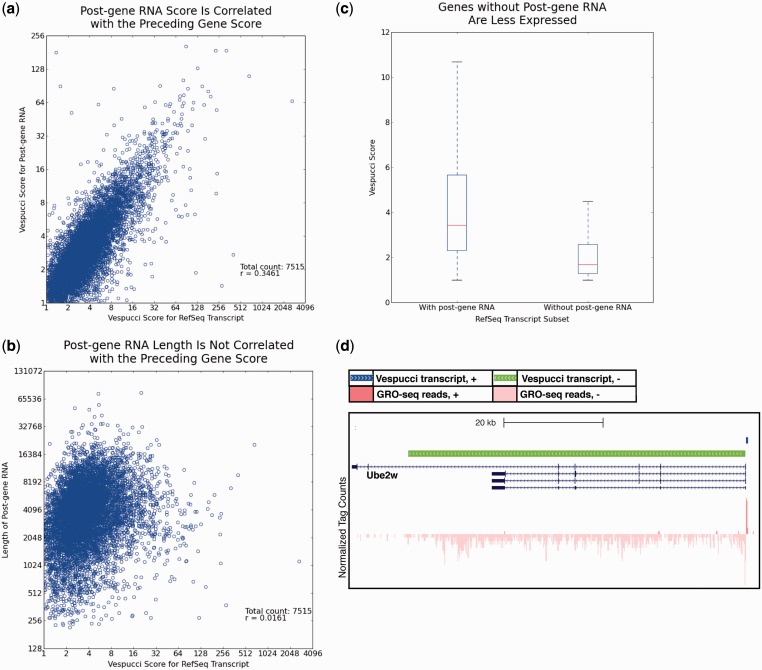
Transcription continues past the annotated 3′ ends of most genes. (**a**) The expression levels of transcripts immediately following the 3′ ends of RefSeq sequences are correlated with those of the preceding RefSeq transcripts as measured with Vespucci scores. (**b**) The length that transcription carries past the 3′ end has a weak but positive correlation with the expression level of the preceding RefSeq transcript as measured with Vespucci scores. (**c**) The 16% of RefSeq transcripts are not found to have post-gene RNA according to Vespucci. These RefSeq transcripts tend to have much lower expression levels as measured with Vespucci scores than the 84% of transcripts that do continue past their annotated 3′ ends. (**d**) In addition to having low expression levels, many of the RefSeq transcripts without post-gene RNA are notable in that the transcript called by Vespucci does not reach the annotated 3′ end of the gene, as is the case with the Ube2w gene here.

Particularly interesting to us was the set of transcripts that continued past the annotated 3′ ends of RefSeq transcripts. Closer inspection of this subset revealed that the vast majority (7346; 84%) of the ∼8700 expressed RefSeq transcripts did not terminate at the annotated TTS, but instead continued for some length afterward (Figure 1d). The expression levels of these ‘post-gene RNAs’ were well correlated with the RefSeq transcripts they followed (Figure 4a), but the lengths of the post-gene RNAs were not determined by the lengths of the associated RefSeq transcripts (Supplementary Figure S3a) or the expression level of the associated RefSeq transcripts (Figure 4b, Supplementary Figure S3b).

We next sought to determine why 16% of the expressed RefSeq transcripts did not continue past the annotated TTS. Of the 1396 RefSeq transcripts with no associated post-gene transcripts, 157 (11%) were labeled as rRNA rather than mRNA by RefSeq. The remaining mRNA had significantly lower expression levels than the set of RefSeq transcripts with post-gene RNA (Figure 4c, Supplementary Figure S3c), and transcription of these genes often did not reach the annotated TTS at all (Figure 4d).

Given this difference in expression level, we next filtered the set of ∼8700 expressed RefSeq transcripts down to the set of 6913 mRNA transcripts that do not stop before the annotated 3′ end of the gene (84% of the 8231 expressed RefSeq mRNAs). Remarkably, 6305 (91%) of this set had associated post-gene RNA, indicating that the annotated TTSs of RefSeq genes greatly underestimate the extent of RNA transcription at these sites.

### Confirming results in human cells

To confirm the extensibility of the results obtained in the macrophage data, we used Vespucci to analyze human GRO-sequencing data from MCF-7 cells from two separate studies (2,12). We used the same parameter values to ensure that the default values selected were not applicable only to murine data. In the human cell line, a higher percentage of transcripts were unannotated than in the mouse cells (Supplementary Figure S2d). The distribution of types of unannotated transcripts was surprisingly similar between the two MCF-7 cell studies (Supplementary Figure S2e, left versus right panels). Fewer transcripts were called as eRNA as compared with the murine data; this is most likely due to the fact that there is relatively little histone data available in MCF-7 cells, and the publicly available H3K4me2 data used here (19) was less deep than the mouse H3K4me1 data used earlier in text. A larger fraction of the human unannotated transcripts remained unassigned to a known category of RNA. Manual inspection of these transcripts revealed that many overlapped with LINE, SINE and LTR elements identified by the RepeatMasker database (Repeat Library 20120124, accessed at http://www.repeatmasker.org). We used Vespucci to annotate the remaining transcripts that overlapped with LINE elements and found that more than half of the remaining transcripts occurred at LINE elements. This corroborated recent reports of widespread transcription at retrotransposons being associated with oncogenesis (29–31). As a whole, these results indicated that both the default parameter values set in Vespucci and the analysis performed for mouse macrophages earlier in text could be repeated in data from multiple cell types, species and labs.

### Benchmarking Vespucci

#### RefSeq benchmarking

**Figure 5.**
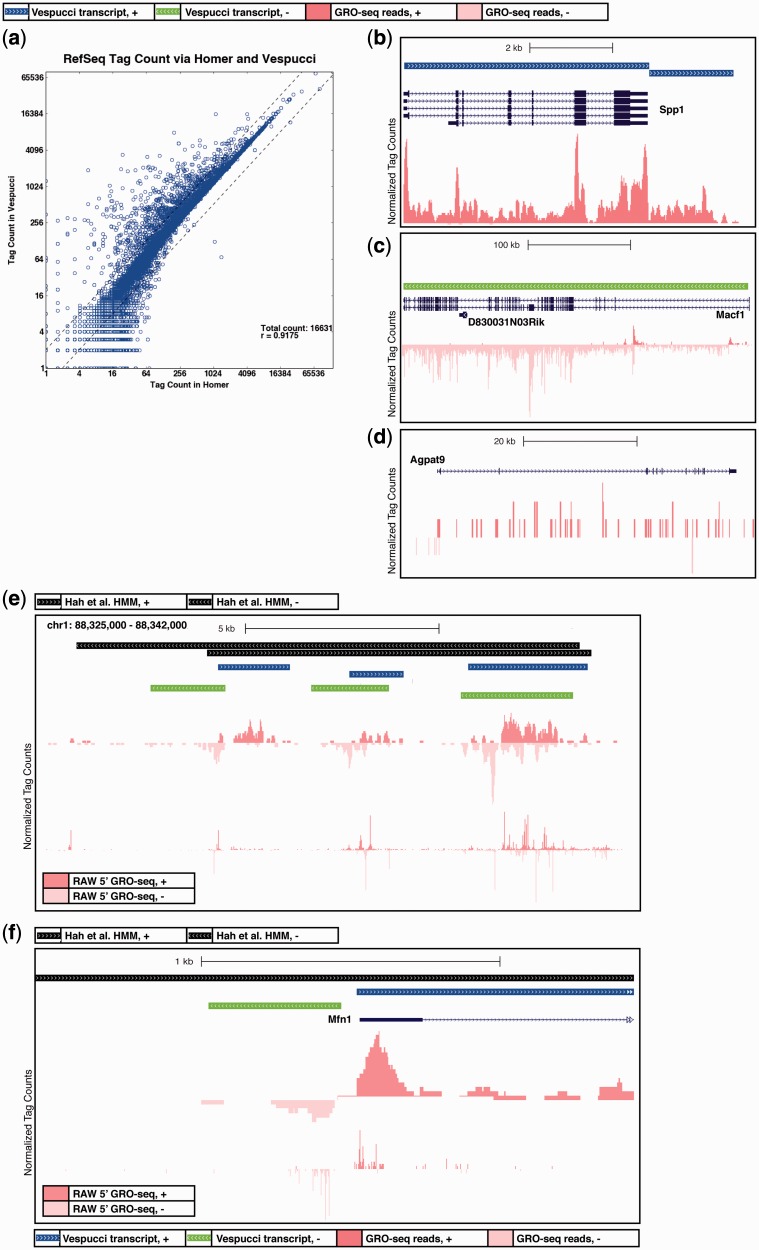
Vespucci retrieves RefSeq expression levels without losing non-coding RNAs. (**a**) RefSeq identifiers can be used to compare the tag counts determined by Vespucci at RefSeq genes with the tag counts determined by the HOMER software, which uses a gene-centric approach to sum GRO-seq tags over known genes. The correlation between tag counts is generally good, with deviations from the diagonal attributable to three primary categories of transcripts: (**b**) Vespucci does not segment transcripts at alternative isoforms, but returns the tags for the whole transcript for each contained isoform. In contrast, HOMER tallies tags within the precise boundaries of each isoform, resulting in discrepancies between the two methods at shorter isoforms, such as the Spp1 gene seen here; (**c**) as with multiple isoforms, overlapping genes are not segmented by Vespucci, and the tag count for the entire transcript covering Macf1 is associated with the short gene that is overlapping, D830031N03Rik; and (**d**) genes that have few dispersed tags that cannot be adequately merged yield several smaller transcripts according to Vespucci, whereas HOMER implicitly joins them and counts all that fall along the body of the gene regardless of continuity of transcription. (**e**) The HMM described by Hah *et al.* identifies transcripts using a two-state model that calls regions transcribed (black bars) or untranscribed. The HMM identifies many fewer transcripts than Vespucci, in part, because it merges together transcripts called as distinct by Vespucci. Here, three pairs of bi-directional RNAs that are identified as two single units by the HMM. The bottom track shows data from previously published 5′-GRO-seq, a method that detects nascent RNA with a 5′ 7-methylguanylated cap. This method identifies start sites of nascent RNAs genome-wide. The data here, from RAW macrophages, show that Vespucci captures more accurately the separately initiated transcripts. (**f**) Similarly, some transcripts are called by Vespucci at expression levels too low for the HMM. Here, a paRNA is identified by Vespucci but not the HMM. The bottom track again shows 5′-GRO-seq from RAW macrophages, where the paRNA start site can be clearly seen.

The two exceptions made for RefSeq annotations noted above allow for consistency with the widely maintained standard of counting tags over RefSeq regions. We compared the shared set of RefSeq transcripts identified by Vespucci with those identified by the ‘analyzeRNA’ method available in the HOMER (7) software package (Figure 5a) and found a high degree of correlation (*r* = 0.92). There are two systematic differences that account for the tag count discrepancies between the two data sets: HOMER sums tags for each RefSeq transcript separately, whereas Vespucci stitches over overlapping genes and isoforms and assigns the total tag count for the longest joined transcript to each associated RefSeq transcript (Figure 5b and c); and HOMER does not require continuity across long transcripts, and consequently counts tags that are missed by Vespucci when genes are too sparse to be adequately stitched together (Figure 5d). Notably, these discrepancies primarily affect transcripts that are difficult to interpret using GRO-sequencing data, as it is unclear how to divide up tag counts across overlapping transcripts (Figure 5b and c) or long, low-level transcripts (Figure 5d). Thus, with these two exceptions made for RefSeq transcripts, Vespucci produces transcripts comparable to existing annotations and methods of analysis.

#### Benchmarking against a HMM

Hah *et al.* (2) describe an HMM that determines regions of transcription from GRO-seq data using a two-state model. To assess the relevance of the transcripts found by Vespucci to those found by the Hah *et al.* HMM, we trained an HMM using the macrophage GRO-sequencing data described earlier in text. Parameters were optimized based on the prescribed procedure for the HMM, which relies on the sum of two errors: (i) the fraction of RefSeq transcripts that are broken apart by the called GRO-seq transcripts (Supplementary Figure S4a) and (ii) the fraction of GRO-seq transcripts that merge two or more RefSeq transcripts (Supplementary Figure S4b). Using these criteria, the minimum summed error (12.7%) was achieved with a negative log transition probability of 100 and a shape parameter of 5. These parameters were used in the model compared directly with Vespucci, though similar results were achieved using the parameters selected by the optimization performed by Hah *et al.* (200 and 5, respectively).

We calculated the Vespucci’s summed error according to the procedure used for the Hah *et al.* HMM and found the error to be 1.8%, or one-sixth of the comparable error for the HMM. This lower error serves to underline the advantage of using RefSeq boundaries to inform transcript identification, as it prevents Vespucci from breaking apart or merging together known regions of transcription. Notably, if Vespucci is used with the same parameters but without any prior knowledge of RefSeq regions, the summed error is about three times that of the optimized Hah *et al.* HMM (37.2%). This highlights both the advantage Vespucci gains by integrating with existing databases, and the fact that the default parameter set is designed to avoid overmerging unannotated regions of transcription. If a user desires to retrieve RefSeq transcripts without prior knowledge of RefSeq, a larger MAX_EDGE parameter may be used to achieve a lower error. With the macrophage data and no assumption of RefSeq boundaries, Vespucci with a MAX_EDGE parameter of 5000 yielded a summed error rate of 12.2% (Supplementary Figure S4c), just below that achieved with the Hah *et al.* HMM.

Given the designed use of Vespucci, the real question of performance comes with transcript calling over unannotated regions of transcription. Whereas Vespucci identifies 24 428 transcripts above a threshold score of 1 that do not overlap same strand with a RefSeq transcript, the HMM identifies 9374. Closer inspection of this discrepancy reveals that the HMM is more likely to merge together transcripts Vespucci calls as separate (Figure 5e) and less likely to call transcripts when GRO-seq expression levels are low (Figure 5f). Further, we compared the calls made by Vespucci and the HMM with the previously published 5′-GRO-seq data (10), and Vespucci more accurately captured the multiplicity of short transcripts associated with distinct transcripts than the HMM (Figure 5e and f). To quantify this merging or missing of transcripts by the HMM, we calculated the same two error rates described earlier in text for the HMM as compared with Vespucci, and found that 35.4% (7026) of the HMM transcripts are broken up by Vespucci transcripts, whereas 1.3% (451) of Vespucci transcripts are broken up by HMM transcripts. Notably, changing the parameters of the HMM might result in higher sensitivity identification of transcripts, but only at the expense of reliable calling of RefSeq genes.

## DISCUSSION

GRO-sequencing reveals transcriptional dynamics at a genome-wide scale, and thus has the power to give unique and novel insight into the regulation of cellular processes. Taking full advantage of this new data source requires combining disparate data sets and identifying within them transcripts of interest. The system introduced here makes this possible by providing a framework for analyzing GRO-sequencing data at both a general level and in great detail. Further, Vespucci allows for easy integration of many different types of sequencing data, which, when taken together, greatly increase the information gained from each single data type.

In this study, we apply Vespucci to annotate nascent RNA transcripts defined by GRO-sequencing data obtained from primary mouse macrophages and a human breast cancer cell line. This analysis yields a comprehensive list of contiguous nascent transcription units derived from both promoters and enhancers throughout the genome. The Vespucci output provides genomic location, score, nearest gene and expression level in various sequencing runs of interest. By enabling the quantification of GRO-sequencing data, we add it to the set of sequencing-based methods that can be reliably leveraged to investigate a wide array of biological questions. In the current study, we demonstrate the use of Vespucci to identify novel transcripts of interest, such as the long non-coding transcript near Gm14461, and characterize the length and expression values of enhancer-associated RNAs. As each cell type contains a specific complement of enhancers that specify its identity and functional potential, Vespucci will be a valuable tool for annotation of cell-specific eRNAs. In addition, Vespucci quantifies the extent of nascent transcription beyond the annotated 3′ ends of genes defined by the site of polyadenylation. This information may be useful in evaluating mechanisms and regulation of transcriptional termination.

One shortfall of the current system is that the parameter defining acceptable gap distance between reads associated with the same transcript must be set heuristically, dependent on the needs of the user. Ideally, the parameters to identify transcriptional units from reads would be set to minimize errors against a gold standard of transcriptional units. At this time, no such standard for GRO-sequencing data exists. In the current study, we were able to approximate a gold standard using 5′-GRO-sequencing data, and thus with Vespucci we hope to take the first step toward defining such a gold standard by providing a method and a framework for transcript identification.

Vespucci extends beyond GRO-sequencing data, too; once the database is set up, it is straightforward to add data from ChIP-sequencing runs, external databases, known motifs, single nucleotide polymorphisms or any other data of interest that can be expressed within genomic coordinate space. In the current study, we demonstrated the integration of data on retrotransposons with the use of LINE data in analyzing MCF-7 cells. Similarly, one might integrate data on repeat regions and mappability; it is possible to load in genomic coordinates of regions of the genome that preclude uniquely mapped reads and then allow the merging of transcripts to automatically ignore those regions. In the current implementation, we do not include this functionality, as it was found to yield too many spurious results. However, if a particular application prefers inclusiveness in transcript merging, automatically covering across repeat regions can be incorporated into the system. This is just one example of the extensibility of the system, demonstrating that Vespucci allows for integration of many types of genomic data and sequencing samples, making more feasible analyses that cut across the whole breadth of samples and data sets available to a laboratory.

## SUPPLEMENTARY DATA

Supplementary Data are available at NAR Online.

## FUNDING

National Institutes of Health [CA17390-01 to C.K.G.]. Funding for open access charge: National Institutes of Health.

*Conflict of interest statement*. None declared.

## Supplementary Material

Supplementary Data

## References

[1] Core LJ, Waterfall JJ, Lis JT (2008). Nascent RNA sequencing reveals widespread pausing and divergent initiation at human promoters. Science.

[2] Hah N, Danko CG, Core L, Waterfall JJ, Siepel A, Lis JT, Kraus WL (2011). A rapid, extensive, and transient transcriptional response to estrogen signaling in breast cancer cells. Cell.

[3] Wang D, Garcia-Bassets I, Benner C, Li W, Su X, Zhou Y, Qiu J, Liu W, Kaikkonen MU, Ohgi KA (2011). Reprogramming transcription by distinct classes of enhancers functionally defined by eRNA. Nature.

[4] Kaikkonen MU, Spann NJ, Heinz S, Romanoski CE, Allison KA, Stender JD, Chun HB, Tough DF, Prinjha RK, Benner C (2013). Remodeling of the enhancer landscape during macrophage activation is coupled to enhancer transcription. Mol. Cell.

[5] Kaikkonen MU, Lam MTY, Glass CK (2011). Non-coding RNAs as regulators of gene expression and epigenetics. Cardiovasc. Res..

[6] Pruitt KD, Tatusova T, Brown GR, Maglott DR (2012). NCBI Reference Sequences (RefSeq): current status, new features and genome annotation policy. Nucleic Acids Res..

[7] Heinz S, Benner C, Spann N, Bertolino E, Lin YC, Laslo P, Cheng JX, Murre C, Singh H, Glass CK (2010). Simple combinations of lineage-determining transcription factors prime cis-regulatory elements required for macrophage and B cell identities. Mol. Cell.

[8] Trapnell C, Williams BA, Pertea G, Mortazavi A, Kwan G, van Baren MJ, Salzberg SL, Wold BJ, Pachter L (2010). Transcript assembly and quantification by RNA-Seq reveals unannotated transcripts and isoform switching during cell differentiation. Nat. Biotechnol..

[9] Djebali S, Davis CA, Merkel A, Dobin A, Lassmann T, Mortazavi A, Tanzer A, Lagarde J, Lin W, Schlesinger F (2012). Landscape of transcription in human cells. Nature.

[10] Lam MTY, Cho H, Lesch HP, Gosselin D, Heinz S, Tanaka-Oishi Y, Benner C, Kaikkonen MU, Kim AS, Kosaka M (2013). Rev-Erbs repress macrophage gene expression by inhibiting enhancer-directed transcription. Nature.

[11] Lai F, Orom UA, Cesaroni M, Beringer M, Taatjes DJ, Blobel GA, Shiekhattar R (2013). Activating RNAs associate with Mediator to enhance chromatin architecture and transcription. Nature.

[12] Li W, Notani D, Ma Q, Tanasa B, Nunez E, Chen AY, Merkurjev D, Zhang J, Ohgi K, Song X (2013). Functional roles of enhancer RNAs for oestrogen-dependent transcriptional activation. Nature.

[13] Melo CA, Drost J, Wijchers PJ, van de Werken H, de Wit E, Oude Vrielink JA, Elkon R, Melo SA, Leveille N, Kalluri R (2013). eRNAs are required for p53-dependent enhancer activity and gene transcription. Mol. Cell.

[14] Kim TK, Hemberg M, Gray JM, Costa AM, Bear DM, Wu J, Harmin DA, Laptewicz M, Barbara-Haley K, Kuersten S (2010). Widespread transcription at neuronal activity-regulated enhancers. Nature.

[15] De Santa F, Barozzi I, Mietton F, Ghisletti S, Polletti S, Tusi BK, Muller H, Ragoussis J, Wei CL, Natoli G (2010). A large fraction of extragenic RNA pol II transcription sites overlap enhancers. PLoS Biol..

[16] Bernstein BE, Birney E, Dunham I, Green ED, Gunter C, Snyder M (2012). An integrated encyclopedia of DNA elements in the human genome. Nature.

[17] Ingolia NT, Ghaemmaghami S, Newman JRS, Weissman JS (2009). Genome-wide analysis *in vivo* of translation with nucleotide resolution using ribosome profiling. Science.

[18] Langmead B, Salzberg SL (2012). Fast gapped-read alignment with Bowtie 2. Nat. Methods.

[19] Tsai WW, Wang Z, Yiu TT, Akdemir KC, Xia W, Winter S, Tsai CY, Shi X, Schwarzer D, Plunkett W (2010). TRIM24 links a non-canonical histone signature to breast cancer. Nature.

[20] Langmead B, Trapnell C, Pop M, Salzberg SL (2009). Ultrafast and memory-efficient alignment of short DNA sequences to the human genome. Genome Biol..

[21] Mortazavi A, Williams BA, McCue K, Schaeffer L, Wold B (2008). Mapping and quantifying mammalian transcriptomes by RNA-Seq. Nat. Methods.

[22] Katz Y, Wang ET, Airoldi EM, Burge CB (2010). Analysis and design of RNA sequencing experiments for identifying isoform regulation. Nat. Methods.

[23] Kim TK, Hemberg M, Gray JM, Costa AM, Bear DM, Wu J, Harmin DA, Laptewicz M, Barbara-Haley K, Kuersten S (2010). Widespread transcription at neuronal activity-regulated enhancers. Nature.

[24] Li H, Handsaker B, Wysoker A, Fennell T, Ruan J, Homer N, Marth G, Abecasis G, Durbin R (2009). The sequence alignment/map format and SAMtools. Bioinformatics.

[25] Ji X, Zhou Y, Pandit S, Huang J, Li H, Lin CY, Xiao R, Burge CB, Fu XD (2013). SR proteins collaborate with 7SK and promoter-associated nascent RNA to release paused polymerase. Cell.

[26] Mituyama T, Yamada K, Hattori E, Okida H, Ono Y, Terai G, Yoshizawa A, Komori T, Asai K (2009). The Functional RNA Database 3.0: databases to support mining and annotation of functional RNAs. Nucleic Acids Res..

[27] He HH, Meyer CA, Shin H, Bailey ST, Wei G, Wang Q, Zhang Y, Xu K, Ni M, Lupien M (2010). Nucleosome dynamics define transcriptional enhancers. Nat. Genet..

[28] Heintzman ND, Stuart RK, Hon G, Fu Y, Ching CW, Hawkins RD, Barrera LO, Van Calcar S, Qu C, Ching KA (2007). Distinct and predictive chromatin signatures of transcriptional promoters and enhancers in the human genome. Nat. Genet..

[29] Kaer K, Speek M (2013). Retroelements in human disease. Gene.

[30] Mandal AK, Pandey R, Jha V, Mukerji M (2013). Transcriptome-wide expansion of non-coding regulatory switches: evidence from co-occurrence of Alu exonization, antisense and editing. Nucleic Acids Res..

[31] Tang RB, Wang HY, Lu HY, Xiong J, Li HH, Qiu XH, Liu HQ (2005). Increased level of polymerase III transcribed Alu RNA in hepatocellular carcinoma tissue. Mol. Carcinog..

