# Supplementary material for: Vespucci: a system for building annotated databases of nascent transcripts
Source: Nucleic Acids Res. 2013 Dec 4;42(4):2433–47. doi: 10.1093/nar/gkt1237 (PMC3936758; doi:10.1093/nar/gkt1237)
Supplement: Supplementary Data [file supp_42_4_2433__index.html]

Vespucci: a system for building annotated databases of nascent transcripts — Vespucci: a system for building annotated databases of nascent transcripts — Supplementary Data 

# Vespucci: a system for building annotated databases of nascent transcripts

## Supplementary Data

files

**Files in this Data Supplement:**

- Supplementary Data - pdf file
- Supplementary Data - pdf file
- Supplementary Data - pdf file
