# Supplementary material for: Vespucci: a system for building annotated databases of nascent transcripts
Source: Nucleic Acids Res. 2013 Dec 4;42(4):2433–47. doi: 10.1093/nar/gkt1237 (PMC3936758; doi:10.1093/nar/gkt1237)

Figure S1 - Glass

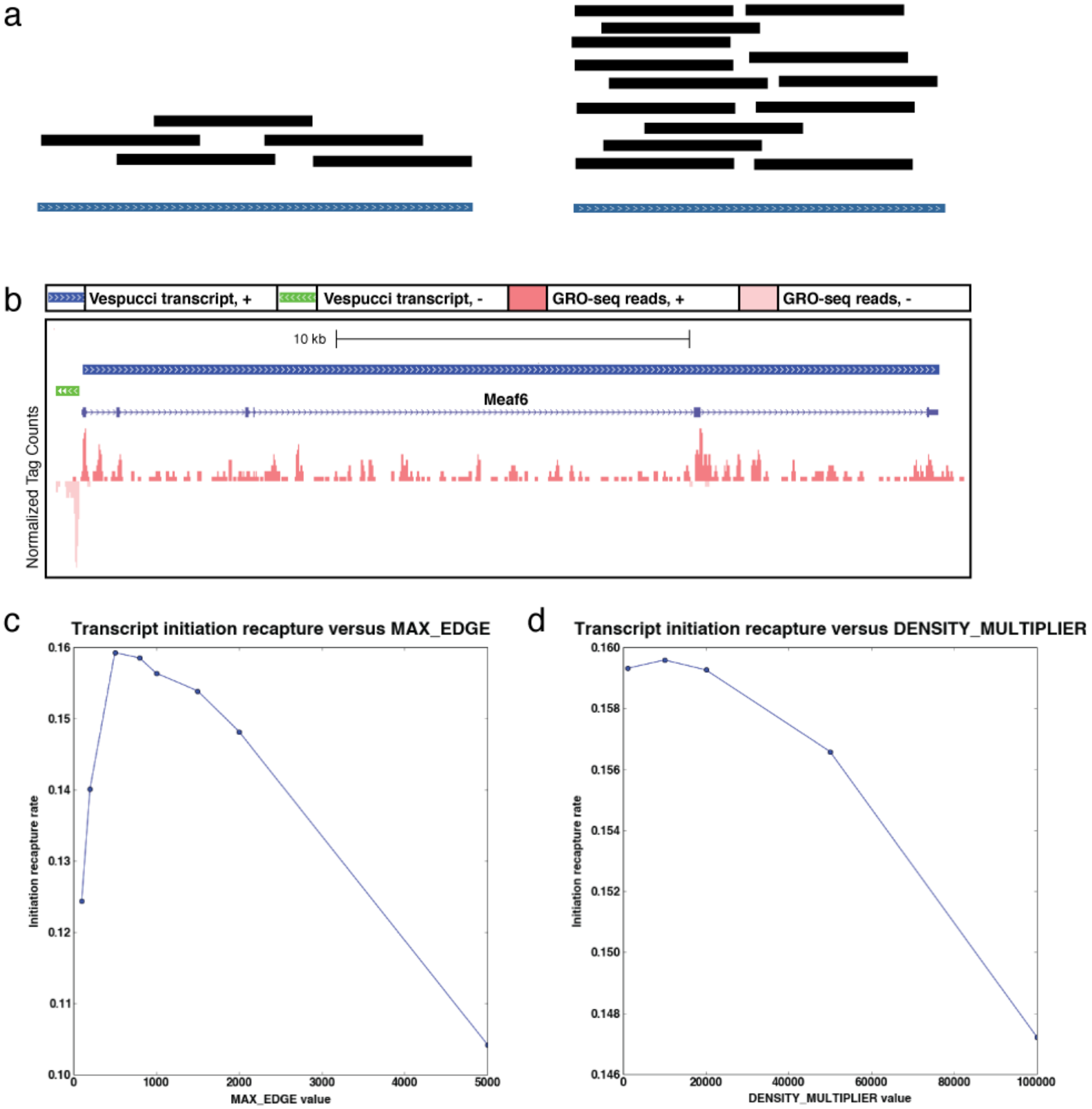

d

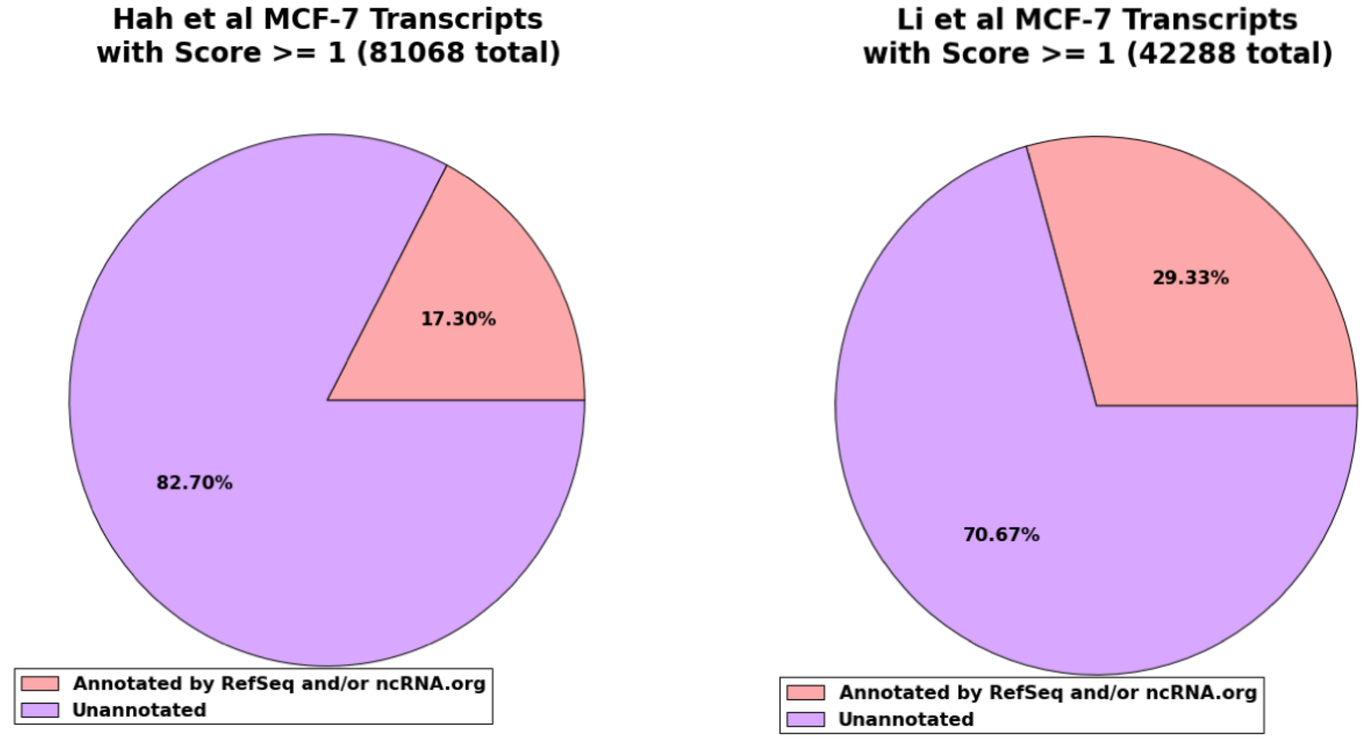

Hah et al MCF-7 Transcripts with Score >= 1 (81068 total)
17.30%
82.70%
Annotated by RefSeq and/or ncRNA.org
Unannotated
Li et al MCF-7 Transcripts with Score >= 1 (42288 total)
29.33%
70.67%
Annotated by RefSeq and/or ncRNA.org
Unannotated

e

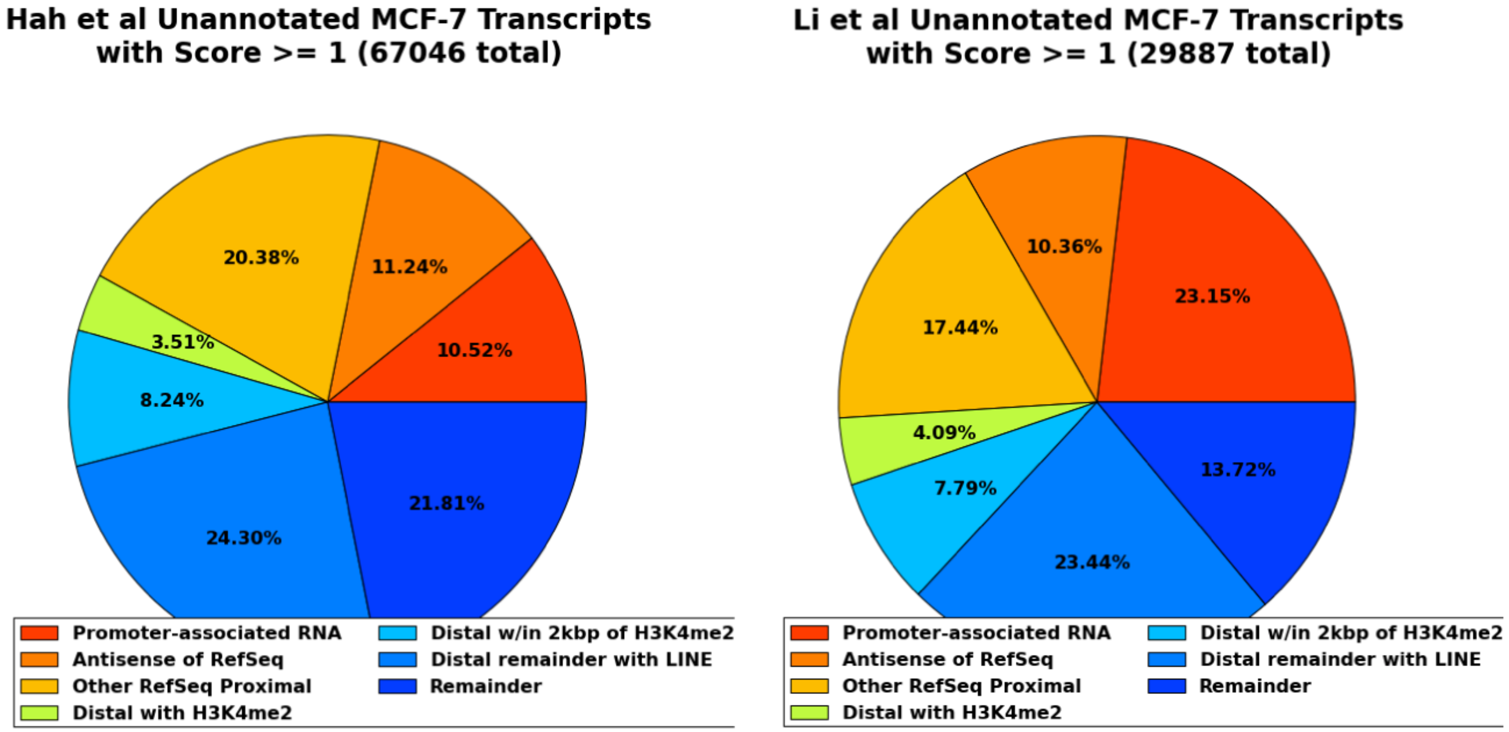

Hah et al Unannotated MCF-7 Transcripts with Score >= 1 (67046 total)
20.38%
11.24%
3.51%
10.52%
8.24%
21.81%
24.30%
Promoter-associated RNA
Antisense of RefSeq
Other RefSeq Proximal
Distal with H3K4me2
Distal w/in 2kbp of H3K4me2
Distal remainder with LINE
Remainder
Li et al Unannotated MCF-7 Transcripts with Score >= 1 (29887 total)
10.36%
23.15%
17.44%
4.09%
13.72%
7.79%
23.44%
Promoter-associated RNA
Antisense of RefSeq
Other RefSeq Proximal
Distal with H3K4me2
Distal w/in 2kbp of H3K4me2
Distal remainder with LINE
Remainder

Figure S2 - Glass

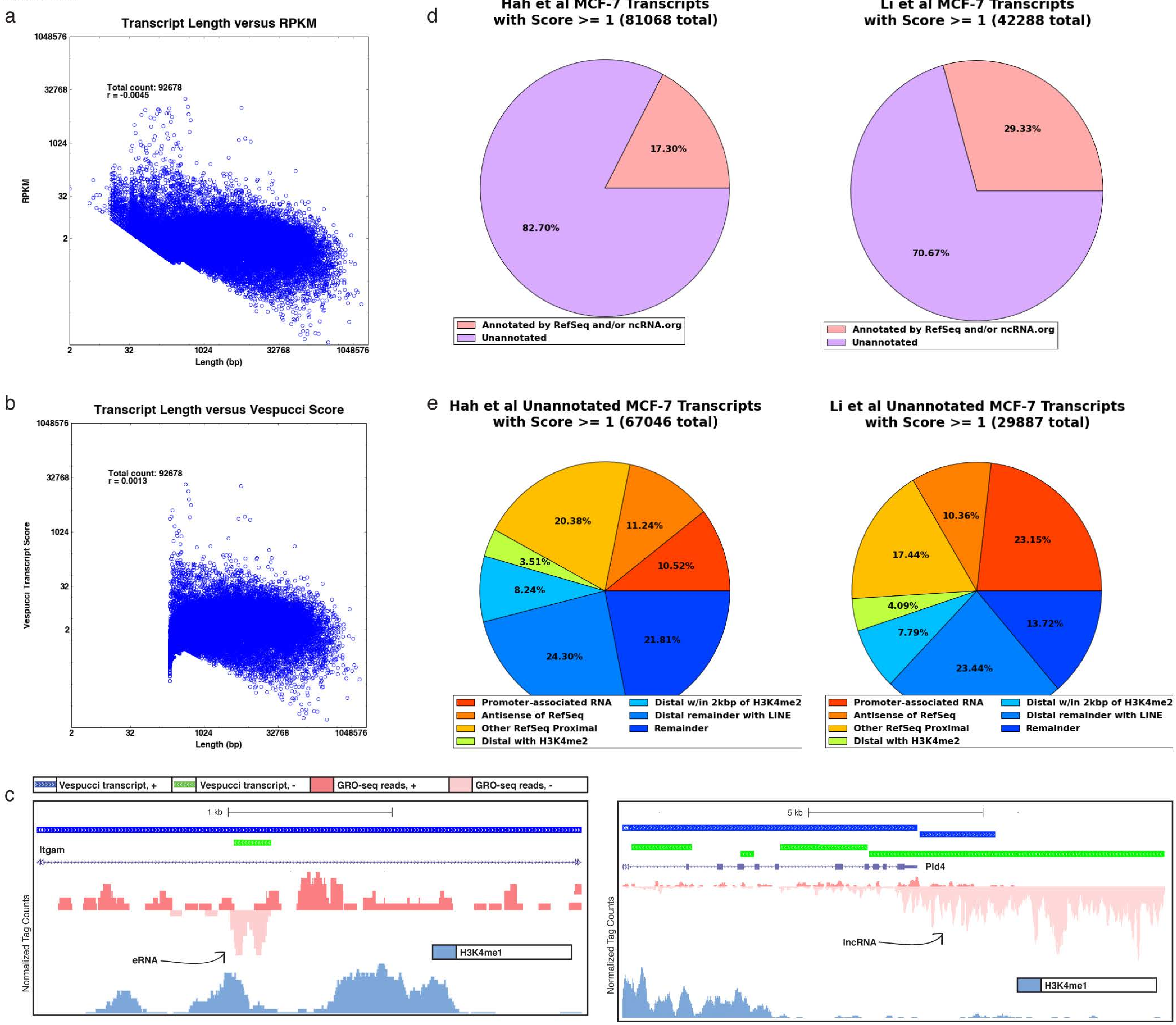

Figure S3 - Glass

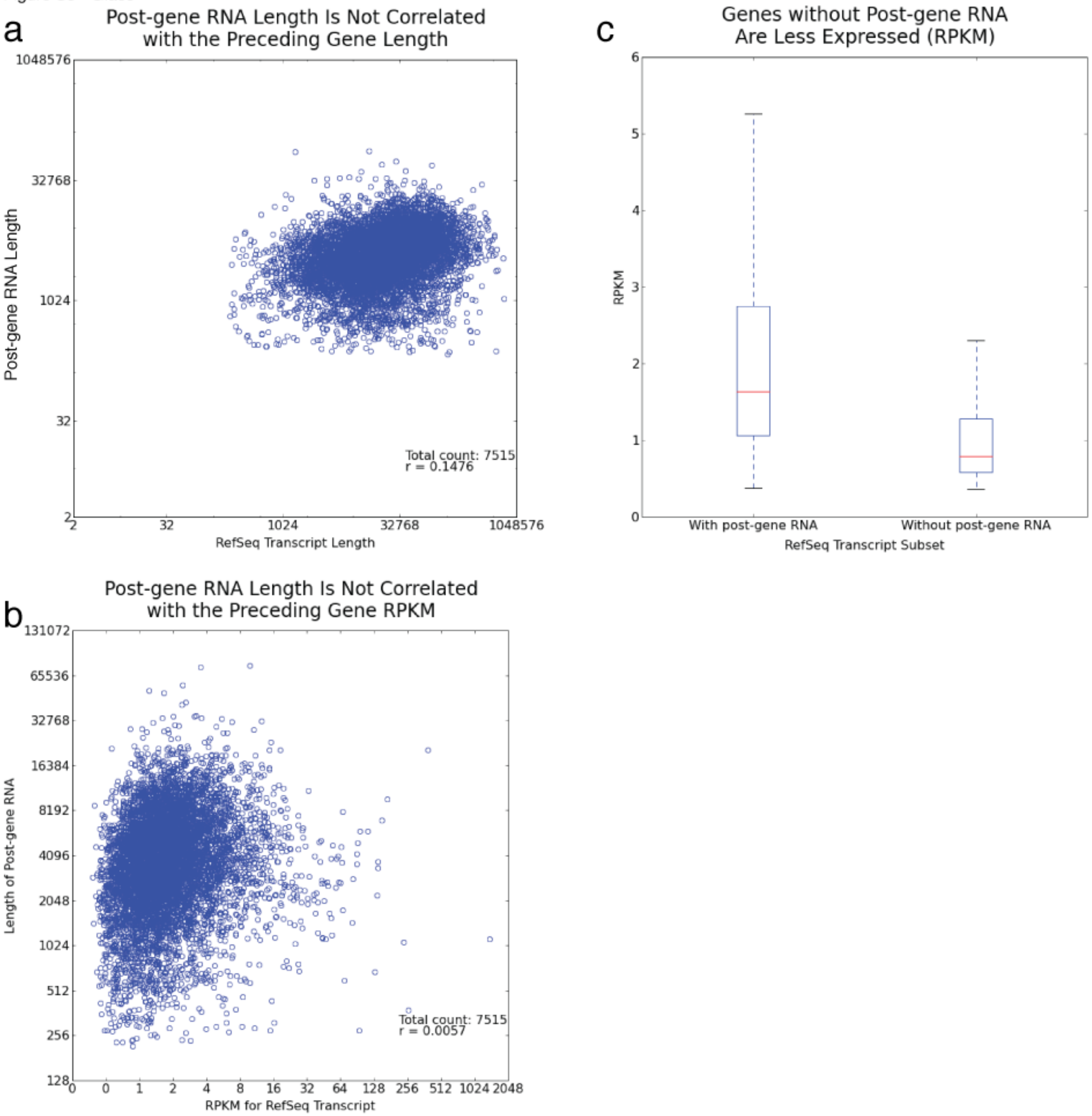

Figure S4- Glass

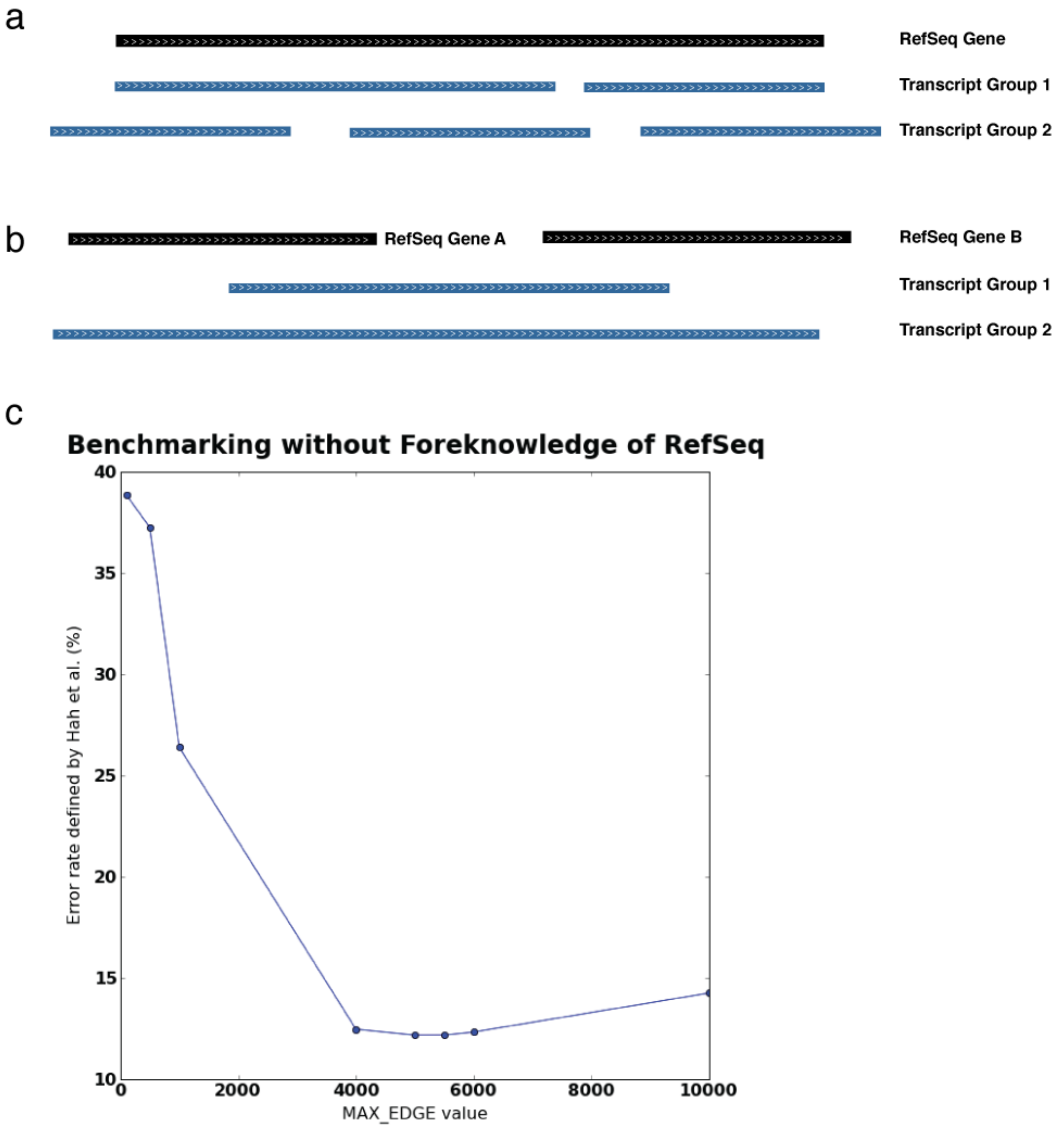

Supplement: Supplementary Data [file supp_gkt1237_nar-02813-n-2013-File007.pdf]
