# Supplementary material for: Vespucci: a system for building annotated databases of nascent transcripts
Source: Nucleic Acids Res. 2013 Dec 4;42(4):2433–47. doi: 10.1093/nar/gkt1237 (PMC3936758; doi:10.1093/nar/gkt1237)
Supplement: Supplementary Data [file supp_gkt1237_nar-02813-n-2013-File008.pdf]

Figure S1

Principles of Vespucci analysis. a. Schematic of read pile-ups, showing regions that are continuously tiled on the left and right. These two regions have different densities, with the right having a higher read-per-base-pair value than the left. These differences in density can advise whether to consider two separate tiled regions as two separately regulated transcripts or as a single unit of transcription. **b.** Vespucci takes into account known RefSeq annotations when merging transcripts. This allows the unification of genic transcripts that exhibit numerous gaps due to low expression levels but are known to be continuous, as is the case with the Meaf6 gene here. The blue bar demarcating the transcript generated extends the full length of the gene despite gaps in tags. **c, d.** In order to optimize parameters for the algorithm, we make use of 5'-GRO-seq in RAW macrophages. This data identifies transcription initiation sites genome-wide, and thus ideally there would be only one start site per Vespucci transcript. We thus define an Initiation Recapture Rate (IRR) that measures the extent to which Vespucci aligns with the 5'-GRO-seq data at different parameter settings. In murine macrophages, Vespucci achieved a maximal IRR with a MAX_EDGE of 500 (c) and a DENSITY_MULTIPLIER of 10,000 (d).

Figure S2

Vespucci enables the identification and quantification of numerous RNA species in macrophages. a. RPKM tends to negatively weight long transcripts relative to short transcripts, and thus shows a downward slope in relation to transcript length. **b.** The Vespucci score, on the other hand, is based on the log of the length of the transcript for longer transcripts, which prevents some of the longer transcripts from falling below whatever scoring threshold is set. **c.** Vespucci identifies several different types of transcripts antisense to RefSeq genes, including intragenic enhancers (left) that co-localize with the enhancer mark H3K4me1; and long non-coding RNA species (right) that overlap annotated genes. **d, e.** In order to demonstrate the extensibility of Vespucci to other species, cell types, and experimental hands, we used Vespucci to analyze previously published MCF-7 data from two separate studies. In (d), a smaller fraction of transcripts identified in each of the MCF-7 studies were annotated by the RefSeq or ncRNA databases as compared to murine macrophages. Closer inspection of the unannotated regions (e) demonstrates that Vespucci identifies surprisingly reproducible RNA species in the two MCF-7 studies. A smaller proportion of MCF-7 unannotated transcripts than in the murine macrophages was marked by enhancer-related histone marks, though this may be an artifact of the depth of ChIP-seq data available in these MCF-7 cells. Notably, a large proportion of distal transcripts not otherwise marked overlapped with LINEs, SINEs, and other repeat-rich elements. This finding corroborates previously published research that suggests oncogenesis involves the widespread transcription of retrotransposons.

Figure S3

Transcription continues past the annotated 3' ends of most genes. a. The lengths transcripts immediately following the 3' ends of RefSeq sequences are not correlated with the lengths of the preceding RefSeq transcripts. **b.** The length that transcription carries past the 3' end does not correlate well with the expression level of the preceding

RefSeq transcript as measured with RPKM. **c.** 13% of RefSeq transcripts are not found to have post-gene RNA according to Vespucci. These RefSeq transcripts tend to have much lower expression levels as measured with RPKM than the 87% of transcripts that do continue past their annotated 3' ends.

Figure S4

Hah *et al.* measure two types of error. a. In order to optimize parameters for an HMM, Hah *et al*. measure the fraction of RefSeq transcripts that are broken apart by the called transcripts. Here, transcript groups 1 and 2 break apart a RefSeq gene, and would each increment the measured error by 1/(number of RefSeq transcripts). **b.** The second type of error Hah *et al*. measure is the fraction of called transcripts that merge together RefSeq genes. Here, transcript groups 1 and 2 run together RefSeq Gene A and RefSeq Gene B, and would each increment the measured error by 1/(number of called transcripts). **c.** Comparing Vespucci to RefSeq transcripts using the error defined by Hah *et al*. advantages Vespucci because, in the default configuration, Vespucci has foreknowledge of RefSeq boundaries when defining transcripts. Here, we built transcript datasets using Vespucci without any RefSeq transcript awareness. Even with no foreknowledge of RefSeq boundaries, Vespucci achieved an error rate equivalent to that of the Hah *et al*. HMM with a MAX_EDGE of 5,000.
