# Supplementary material for: Vespucci: a system for building annotated databases of nascent transcripts
Source: Nucleic Acids Res. 2013 Dec 4;42(4):2433–47. doi: 10.1093/nar/gkt1237 (PMC3936758; doi:10.1093/nar/gkt1237)
Supplement: Supplementary Data [file supp_gkt1237_nar-02813-n-2013-File009.pdf]

```sql
-- Transcription per strand
SELECT strand, sum(transcription_end- transcription_start+1)
FROM atlas_mm9_default.atlas_transcript t
GROUP BY t.strand;

-- Total transcripts
SELECT count(t.*) FROM atlas_mm9_default.atlas_transcript t
WHERE t.parent_id IS NULL;

SELECT count(t.*) FROM atlas_mm9_default.atlas_transcript t
WHERE t.score >= 1
AND t.parent_id IS NULL;

SELECT count(t.*) FROM atlas_mm9_default.atlas_transcript t
WHERE t.score >= .75
AND t.parent_id IS NULL;

-- Has Refseq, covers most
SELECT count(distinct t.id) FROM
atlas_mm9_default.atlas_transcript t
JOIN atlas_mm9_default.atlas_transcript_sequence s
ON t.id = s.atlas_transcript_id
WHERE t.score >= 1
AND t.parent_id IS NULL
AND s.major = true;

-- Has Refseq, doesn't cover most
SELECT count(distinct t.id) FROM
atlas_mm9_default.atlas_transcript t
JOIN atlas_mm9_default.atlas_transcript_sequence s
ON t.id = s.atlas_transcript_id
WHERE t.score >= 1
AND t.parent_id IS NULL
AND s.major = false;

-- Has ncRNA
SELECT count(distinct t.id) FROM
atlas_mm9_default.atlas_transcript t
JOIN atlas_mm9_default.atlas_transcript_non_coding nc
ON t.id = nc.atlas_transcript_id
JOIN genome_reference_mm9.non_coding_transcription_region reg
ON nc.non_coding_transcription_region_id = reg.id
JOIN genome_reference_mm9.non_coding_rna rna
ON reg.non_coding_rna_id = rna.id
WHERE t.score >= 1
AND t.parent_id IS NULL;


-- No Refseq or ncRNA
SELECT count(distinct t.id) FROM
atlas_mm9_default.atlas_transcript t
LEFT OUTER JOIN atlas_mm9_default.atlas_transcript_sequence s
ON t.id = s.atlas_transcript_id
LEFT OUTER JOIN atlas_mm9_default.atlas_transcript_non_coding nc
ON t.id = nc.atlas_transcript_id
```

```
WHERE t.score >= 1
AND t.parent_id IS NULL
AND s.id IS NULL
AND nc.id IS NULL;

-- Refseq proximal
SELECT count(distinct t.id) FROM
atlas_mm9_default.atlas_transcript t
LEFT OUTER JOIN atlas_mm9_default.atlas_transcript_sequence s
ON t.id = s.atlas_transcript_id
LEFT OUTER JOIN atlas_mm9_default.atlas_transcript_non_coding nc
ON t.id = nc.atlas_transcript_id
WHERE t.score >= 1
AND t.parent_id IS NULL
AND s.id IS NULL
AND nc.id IS NULL
AND t.distal = false;

-- Promoter associated RNA (paRNA)
SELECT count(distinct t.id) FROM
atlas_mm9_default.atlas_transcript t
LEFT OUTER JOIN atlas_mm9_default.atlas_transcript_sequence s
ON t.id = s.atlas_transcript_id
LEFT OUTER JOIN atlas_mm9_default.atlas_transcript_non_coding nc
ON t.id = nc.atlas_transcript_id
JOIN genome_reference_mm9.sequence_transcription_region reg
ON t.chromosome_id = reg.chromosome_id
AND t.start_end && reg.start_site_1000
AND t.strand != reg.strand
WHERE t.score >= 1
AND t.parent_id IS NULL
AND s.id IS NULL
AND nc.id IS NULL
AND t.distal = false;

-- Promoter associated RNA (paRNA), gene transcribed
SELECT count(distinct t.id) FROM
atlas_mm9_default.atlas_transcript t
LEFT OUTER JOIN atlas_mm9_default.atlas_transcript_sequence s
ON t.id = s.atlas_transcript_id
LEFT OUTER JOIN atlas_mm9_default.atlas_transcript_non_coding nc
ON t.id = nc.atlas_transcript_id
JOIN genome_reference_mm9.sequence_transcription_region reg
ON t.chromosome_id = reg.chromosome_id
AND t.start_end && reg.start_site_1000
AND t.strand != reg.strand
JOIN atlas_mm9_default.atlas_transcript_sequence s2
ON reg.id = s2.sequence_transcription_region_id
AND s2.major = true
JOIN atlas_mm9_default.atlas_transcript t2
ON s2.atlas_transcript_id = t2.id
WHERE t.score >= 1
AND t.parent_id IS NULL
AND t2.score >= 1
AND t2.parent_id IS NULL
```

```
AND s.id IS NULL
AND nc.id IS NULL
AND t.distal = false;

-- Refseq proximal, antisense
SELECT count(distinct t.id) FROM
atlas_mm9_default.atlas_transcript t
LEFT OUTER JOIN atlas_mm9_default.atlas_transcript_sequence s
ON t.id = s.atlas_transcript_id
LEFT OUTER JOIN atlas_mm9_default.atlas_transcript_non_coding nc
ON t.id = nc.atlas_transcript_id
JOIN genome_reference_mm9.sequence_transcription_region reg
ON t.chromosome_id = reg.chromosome_id
AND t.start_end && reg.start_end
AND (t.start_end && reg.start_site_1000) = false
AND t.strand != reg.strand
WHERE t.score >= 1
AND t.parent_id IS NULL
AND s.id IS NULL
AND nc.id IS NULL
AND t.distal = false;

-- Refseq proximal, antisense, gene transcribed
SELECT count(distinct t.id) FROM
atlas_mm9_default.atlas_transcript t
LEFT OUTER JOIN atlas_mm9_default.atlas_transcript_sequence s
ON t.id = s.atlas_transcript_id
LEFT OUTER JOIN atlas_mm9_default.atlas_transcript_non_coding nc
ON t.id = nc.atlas_transcript_id
JOIN genome_reference_mm9.sequence_transcription_region reg
ON t.chromosome_id = reg.chromosome_id
AND t.start_end && reg.start_end
AND (t.start_end && reg.start_site_1000) = false
AND t.strand != reg.strand
JOIN atlas_mm9_default.atlas_transcript_sequence s2
ON reg.id = s2.sequence_transcription_region_id
AND s2.major = true
JOIN atlas_mm9_default.atlas_transcript t2
ON s2.atlas_transcript_id = t2.id
WHERE t.score >= 1
AND t.parent_id IS NULL
AND t2.score >= 1
AND t2.parent_id IS NULL
AND s.id IS NULL
AND nc.id IS NULL
AND t.distal = false;

-- Distal
SELECT count(distinct t.id) FROM
atlas_mm9_default.atlas_transcript t
LEFT OUTER JOIN atlas_mm9_default.atlas_transcript_sequence s
ON t.id = s.atlas_transcript_id
LEFT OUTER JOIN atlas_mm9_default.atlas_transcript_non_coding nc
ON t.id = nc.atlas_transcript_id
WHERE t.score >= 1
```

```
AND t.parent_id IS NULL
AND s.id IS NULL
AND nc.id IS NULL
AND t.distal = true;

-- Distal with H3K4me1
SELECT count(distinct t.id) FROM
atlas_mm9_default.atlas_transcript t
LEFT OUTER JOIN atlas_mm9_default.atlas_transcript_sequence s
ON t.id = s.atlas_transcript_id
LEFT OUTER JOIN atlas_mm9_default.atlas_transcript_non_coding nc
ON t.id = nc.atlas_transcript_id
JOIN thiomac_chipseq.peak_wt_notx_h3k4me1_02_09 p
ON t.chromosome_id = p.chromosome_id
AND t.start_end && p.start_end
WHERE t.score >= 1
AND s.id IS NULL
AND nc.id IS NULL
AND t.distal = true;

-- Distal without H3K4me1
SELECT count(distinct t.id) FROM
atlas_mm9_default.atlas_transcript t
LEFT OUTER JOIN atlas_mm9_default.atlas_transcript_sequence s
ON t.id = s.atlas_transcript_id
LEFT OUTER JOIN atlas_mm9_default.atlas_transcript_non_coding nc
ON t.id = nc.atlas_transcript_id
LEFT OUTER JOIN thiomac_chipseq.peak_wt_notx_h3k4me1_02_09 p
ON t.chromosome_id = p.chromosome_id
AND t.start_end && p.start_end
WHERE t.score >= 1
AND t.parent_id IS NULL
AND s.id IS NULL
AND nc.id IS NULL
AND p.id IS NULL
AND t.distal = true;


-- Within 2kb of H3K4me1
SELECT count(distinct t.id) FROM
atlas_mm9_default.atlas_transcript t
JOIN genome_reference_mm9.chromosome chr
ON t.chromosome_id = chr.id
LEFT OUTER JOIN atlas_mm9_default.atlas_transcript_sequence s
ON t.id = s.atlas_transcript_id
LEFT OUTER JOIN atlas_mm9_default.atlas_transcript_non_coding nc
ON t.id = nc.atlas_transcript_id
JOIN thiomac_chipseq.peak_wt_notx_h3k4me1_02_09 p
ON t.chromosome_id = p.chromosome_id
AND t.start_end && int8range(p."start" - 2000, p."end" + 2000)
WHERE t.score >= 1
AND t.parent_id IS NULL
AND s.id IS NULL
AND nc.id IS NULL
AND t.distal = true;
```

```
-- Post-gene transcripts
SELECT distinct ON (t.id) chr.name,
least(t.transcription_start,t2.transcription_start) as
min_start,
greatest(t.transcription_end, t2.transcription_end) as max_end,
t.*,
t2.id as gene_id,
t2.transcription_start as gene_start,
t2.transcription_end as gene_end,
t2.score as gene_score,
t2.rpkm as gene_rpkm
FROM atlas_mm9_default.atlas_transcript t
JOIN genome_reference_mm9.chromosome chr
ON t.chromosome_id = chr.id
JOIN atlas_mm9_default.atlas_transcript t2
ON t.parent_id = t2.id
WHERE t.score >= 1
AND t2.score >= 1;

-- All expressed Refseq
SELECT * FROM
(SELECT chr.name,
t.transcription_start as min_start,
t.transcription_end as max_end,
t.*, s.*, reg.*, (reg.transcription_end -
reg.transcription_start) as reg_width,
refseq.*,
row_number()
OVER (PARTITION BY t.id ORDER BY reg.transcription_end -
reg.transcription_start DESC)
FROM atlas_mm9_default.atlas_transcript t
JOIN genome_reference_mm9.chromosome chr
ON t.chromosome_id = chr.id
JOIN atlas_mm9_default.atlas_transcript_sequence s
ON t.id = s.atlas_transcript_id
AND s.major = true
JOIN genome_reference_mm9.sequence_transcription_region reg
ON s.sequence_transcription_region_id = reg.id
JOIN genome_reference_mm9.sequence_identifier refseq
ON reg.sequence_identifier_id = refseq.id
WHERE t.score >= 1
AND t.parent_id IS NULL) subq
WHERE row_number = 1;


-- Score, RPKM vectors
SELECT id, score, rpkm, (transcription_end - transcription_start
+ 1) as length FROM atlas_mm9_default.atlas_transcript
ORDER BY id asc;

-- Tag count by Refseq gene
SELECT refseq.id, refseq.sequence_identifier, sum(s.tag_count)
FROM atlas_mm9_default.atlas_transcript t
```

```
JOIN atlas_mm9_default.atlas_transcript_source s
ON t.id = s.atlas_transcript_id
JOIN atlas_mm9_default.atlas_transcript_sequence seq
ON t.id = seq.atlas_transcript_id
JOIN genome_reference_mm9.sequence_transcription_region reg
ON seq.sequence_transcription_region_id = reg.id
JOIN genome_reference_mm9.sequence_identifier refseq
ON reg.sequence_identifier_id = refseq.id
JOIN genome_reference_mm9.sequencing_run run
ON s.sequencing_run_id = run.id
WHERE run.source_table = 'groseq"."tag_wt_notx_05_11'
AND refseq.type = 'mRNA'
GROUP BY refseq.id, refseq.sequence_identifier
ORDER BY refseq.sequence_identifier;
```
